# Plasmids of Carotenoid-Producing *Paracoccus* spp. (*Alphaproteobacteria*) - Structure, Diversity and Evolution

**DOI:** 10.1371/journal.pone.0080258

**Published:** 2013-11-08

**Authors:** Anna Maj, Lukasz Dziewit, Jakub Czarnecki, Miroslawa Wlodarczyk, Jadwiga Baj, Grazyna Skrzypczyk, Dorota Giersz, Dariusz Bartosik

**Affiliations:** Department of Bacterial Genetics, Institute of Microbiology, Faculty of Biology, University of Warsaw, Warsaw, Poland; Institut National de la Recherche Agronomique, France

## Abstract

Plasmids are components of many bacterial genomes. They enable the spread of a large pool of genetic information via lateral gene transfer. Many bacterial strains contain mega-sized replicons and these are particularly common in *Alphaproteobacteria*. Considerably less is known about smaller alphaproteobacterial plasmids. We analyzed the genomes of 14 such plasmids residing in 4 multireplicon carotenoid-producing strains of the genus *Paracoccus* (*Alphaproteobacteria*): *P. aestuarii* DSM 19484, *P. haeundaensis* LG P-21903, *P. marcusii* DSM 11574 and *P. marcusii* OS22. Comparative analyses revealed mosaic structures of the plasmids and recombinational shuffling of diverse genetic modules involved in (i) plasmid replication, (ii) stabilization (including toxin-antitoxin systems of the *relBE*/*parDE*, *tad*-*ata*, *higBA*, *mazEF* and *toxBA* families) and (iii) mobilization for conjugal transfer (encoding relaxases of the Mob_Q_, Mob_P_ or Mob_V_ families). A common feature of the majority of the plasmids is the presence of AT-rich sequence islets (located downstream of *exc1*-like genes) containing genes, whose homologs are conserved in the chromosomes of many bacteria (encoding e.g. RelA/SpoT, SMC-like proteins and a retron-type reverse transcriptase). The results of this study have provided insight into the diversity and plasticity of plasmids of *Paracoccus* spp., and of the entire *Alphaproteobacteria*. Some of the identified plasmids contain replication systems not described previously in this class of bacteria. The composition of the plasmid genomes revealed frequent transfer of chromosomal genes into plasmids, which significantly enriches the pool of mobile DNA that can participate in lateral transfer. Many strains of *Paracoccus* spp. have great biotechnological potential, and the plasmid vectors constructed in this study will facilitate genetic studies of these bacteria.

## Introduction

Bacterial plasmids have a modular structure: their genomes can be separated into several DNA cassettes encoding specific functions. Besides the conserved backbone, composed of genetic modules encoding replication (REP), stabilization and transfer functions, plasmids can contain an additional “genetic load”, which may significantly influence the metabolic properties of any recipient strain. Many plasmids are giant molecules that can even exceed the size of some bacterial chromosomes. Such mega-sized replicons (megaplasmids) are particularly common in *Alphaproteobacteria*. 

*Alphaproteobacteria* constitute interesting models for studying the complexity and diversity of bacterial genomes. Many strains within the genera *Rhizobium*, *Agrobacterium* and *Paracoccus* contain chromosomes, chromids, megaplasmids and sometimes several smaller plasmids (e.g. [1,2]). Analysis of the genomic data collected by The National Center for Biotechnology Information (NCBI) revealed that the sequenced genomes of 240 alphaproteobacterial strains include a total of 315 plasmids. Twenty six of these strains are multi-plasmid containing, with at least five extrachromosomal replicons.

For many years, knowledge of plasmids of the *Alphaproteobacteria* was mainly limited to the *repABC* and *repC* families of replicons, which are specific for megaplasmids of this group of bacteria (*repA and repB* of *repABC* replicons encode partitioning proteins, while *repC* encodes replication initiator) (e.g. [3,4]). Recent studies have revealed the presence of plasmids classified into the *repA* and *repB* families as well as a *dnaA*-like family, encoding replication proteins with similarity to the DnaA proteins involved in the initiation of replication of bacterial chromosomes [1]. Detailed analysis of the REP regions allowed the following incompatibility (inc) groups to be distinguished: (i) 9 groups of *repABC* replicons, (ii) 5 groups of *repA*-family replicons and (iii) 4 groups of *repB*-family replicons [1]. Most of the analyzed REPs are harbored by megaplasmids and much less is known about smaller alphaproteobacterial plasmids. 

Several years ago we initiated a project aimed at identifying and characterizing the pool of mobile DNA in bacteria belonging to the genus *Paracoccus* (*Alphaproteobacteria*). This genus currently comprises 42 species and hundreds of strains (not identified at species level), which are known for their versatile physiological properties and ability to perform a number of different growth modes. We focused our interest on mobile genetic elements (MGE) of *Paracoccus* spp., especially plasmids and transposable elements (TE) (e.g. [5]). As a result of this approach we have identified and analyzed (i) four related *repABC* as well as several pTAV3-type megaplasmids – both groups residing in *P. versutus* UW1 and four strains of *Paracoccus pantotrophus* [6,7], (ii) plasmid pALC1 of *Paracoccus alcaliphilus* JCM 7364, with an iteron-containing replication system [8], (iii) plasmid pMTH1 of *Paracoccus methylutens* DM12, whose genome is predominantly (80%) composed of transposable modules (TMos) [9], (iv) three plasmids of *Paracoccus aminophilus* JCM 7686, whose REP modules were used for the construction of versatile DIY cassettes [10,11], as well as (v) plasmid pWKS1 of *P. pantotrophus* DSM 11072 – the smallest replicon identified so far in *Paracoccus* spp. [12]. 

In this study we identified four plasmid-rich strains of *Paracoccus* spp. Genomic analysis of their plasmids revealed that the genetic organization and structure of many of them differ significantly from that previously described in *Alphaproteobacteria*.

## Results and Discussion

### Plasmids of carotenoid-producing strains of Paracoccus spp.

At the initial stage of this study we analyzed the plasmid content of 22 strains representing 20 *Paracoccus* spp. (listed in Methods section). Plasmid screening revealed that the majority of the tested strains contained megaplasmids (above 100 kb) (data not shown). Only four of the strains (*P. aestuarii* DSM 19484, *P. haeundaensis* LG P-21903, *P. marcusii* DSM 11574 and *P. marcusii* OS22 – all able to produce beta-carotenoid pigments) contained numerous smaller replicons ranging in size from approx. 2.5 kb to 85 kb.

To analyze plasmid diversity in these strains we obtained the nucleotide sequences of 14 randomly selected replicons (listed in Table 1): (i) 5 pAES plasmids of *P. aestuarii* DSM 19484, (ii) 2 pHAE plasmids of *P. haeundaensis* LMG P-21903, (iii) 4 pMARC plasmids of *P. marcusii* DSM 11574 and (iv) 3 pMOS plasmids of *P. marcusii* OS22. 

**Table 1:** Basic characterization of the *Paracoccus* spp. plasmid genomes.

			**GC content (%)**			
**Plasmid**	**Host**	**Size (bp)**	**Plasmid DNA**	**Host DNA**	**Number of ORFs**	**Genetic modules**
pAES1	*P. aestuarii* DSM 19484	2925	64.4	62 [43]	2	REP, MOB
pAES2		4502	57.9		5	REP, R-M
pAES3		5434	51.7		7	REP, TA
pAES4		5850	58.6		9	REP, MOB, TA
pAES7		13,005	60.1		15	REP, MOB, TA(2)
pHAE1	*P. haeundaensis* LG P-21903	5301	58.8	66.9 [44]	4	REP
pHAE2		5777	53.6		5	REP, TA
pMARC1	*P. marcusii* DSM 11574	5122	49.0	66 [45]	6	REP, TA
pMARC2		5789	51.6		6	REP, MOB
pMARC3		10,672	59.0		10	REP, PAR, MOB, TA
pMARC4		15,289	53.4		15	REP, MOB, TA
pMOS2	*P. marcusii* OS22	6410	54.0	66 [45]	5	REP, MOB
pMOS6		7672	63.1		12	REP, MOB, TA
pMOS7		5979	50.0		7	REP, TA

The results of the overall characterization of the plasmids are presented in Table 1. A summary of the distinguished open reading frames (ORFs), including their position, the size of the putative proteins they encode and their closest homologs, is presented in Table S1 in the supplemental material. 

**Figure 1:**
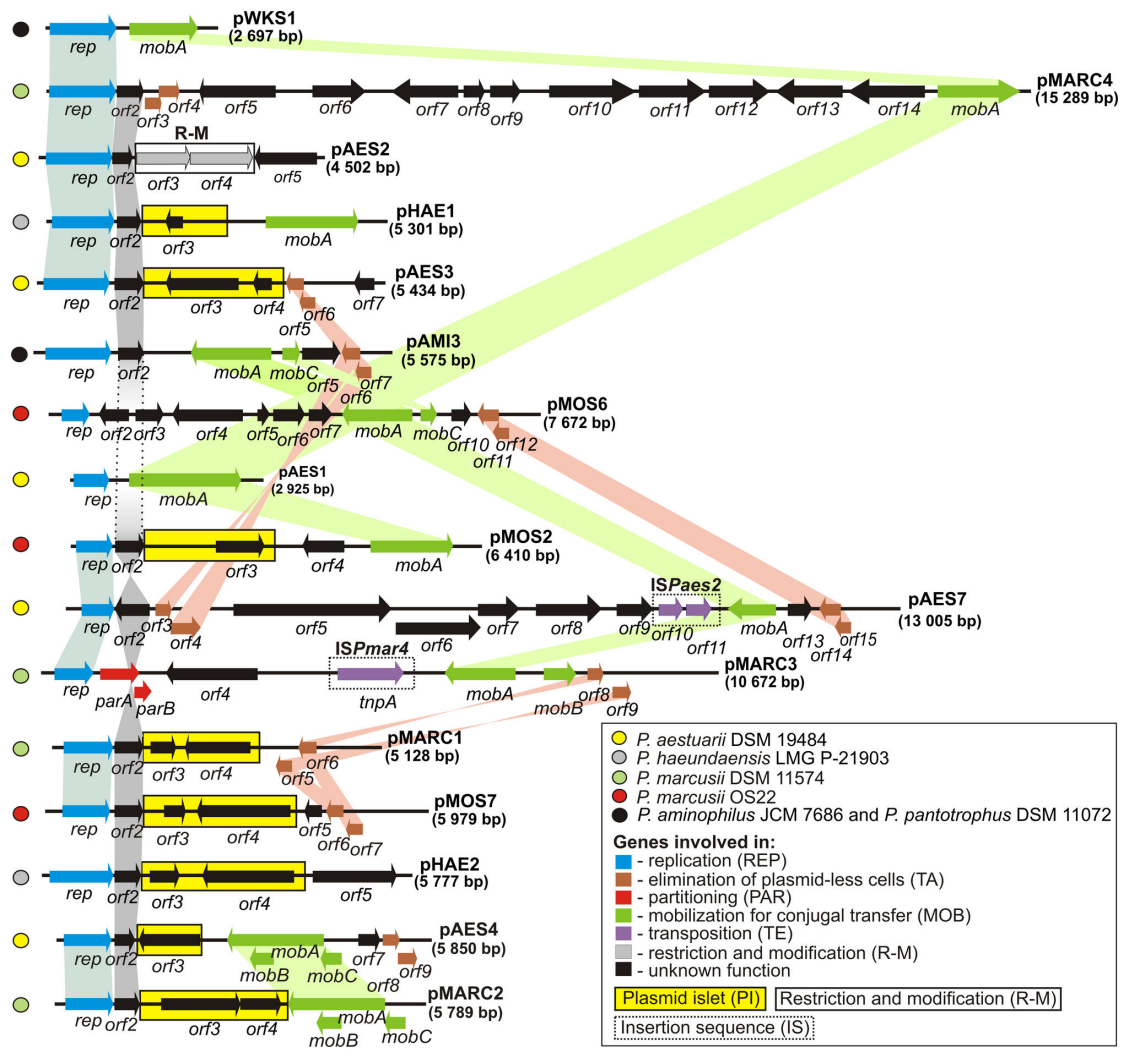
The genetic organization of the *Paracoccus* spp. plasmids analyzed in this study. Arrows indicate the transcriptional orientation of the ORF2. The color-coded keys show the species and strain of origin of each plasmid (circles) and the likely plasmid maintenance/transfer processes in which the genes are involved (squares). Plasmid islets (PI) of lower than average G+C content, insertion sequences (IS) and restriction and modification systems (R-M) are indicated by the use of different boxes (see figure). Shaded areas connect genes of plasmids that encode orthologous proteins. For comparative analysis, two other related plasmids of *Paracoccus* spp. have been included: pAMI3 of *P. aminophilus* JCM 7686 [10] and pWKS1 of *P. pantotrophus* DSM 11072 [12].

Comparative bioinformatic analysis was used to distinguish the plasmid backbones, composed of different combinations of genetic modules responsible for plasmid replication (REP), stabilization (toxin-antitoxin – TA; partitioning – PAR) and mobilization for conjugal transfer (MOB), and accessory genetic information, which may potentially influence the phenotype of the host (Figure 1). Detailed characterization of the predicted modules is presented below.

### Replication modules

Replication systems of the vast majority of plasmids residing in gram-negative bacteria consist of two elements: (i) a gene encoding a Rep protein, which initiates DNA replication, and (ii) the *cis*-required origin (*oriV*; equivalent to chromosomal *oriC*) where replication begins. Most Rep proteins are highly conserved and they can be grouped into several families on the basis of amino acid (aa) sequence similarities. In contrast, *oriV*s are more divergent and they are usually placed in close proximity to the *rep* genes. In many cases, the location of the *oriV*s can be predicted *in silico* by the presence of (i) directly repeated sequences (including iterons), which constitute the Rep protein binding sites (iterons, being key elements in the control of replication initiation, determine plasmid incompatibility [13]), (ii) A+T-rich DNA regions, where strand opening and assembly of host replication initiation factors occurs, and (iii) conserved DNA boxes representing sites of interaction with chromosomally-encoded proteins, e.g. DnaA or integration host factor (IHF) [14]. 

**Figure 2:**
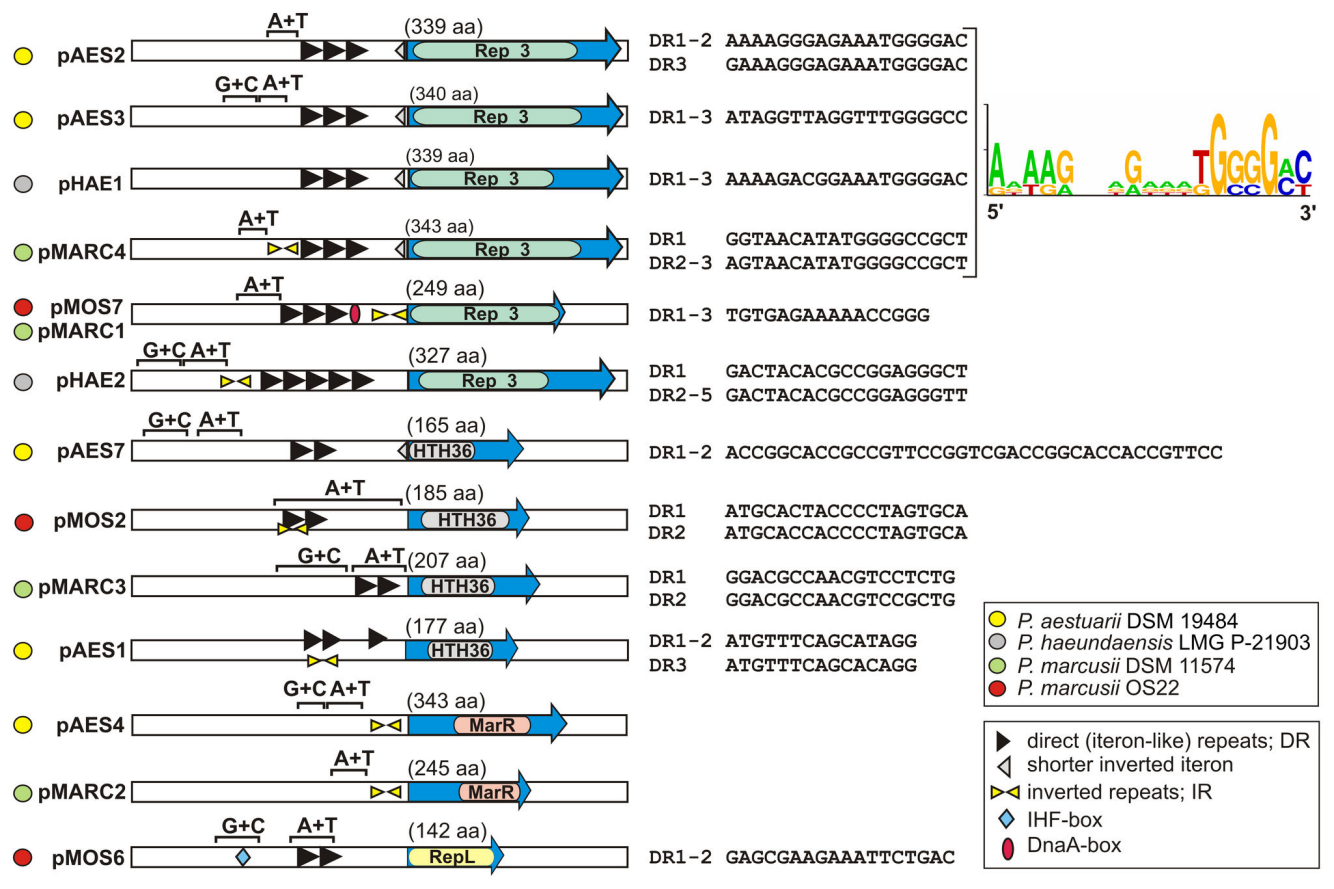
Schematic structure of the REP modules analyzed in this study. The color-coded keys show the species and strain of origin of each plasmid (circles) and identified direct repeats (DRs), inverted repeats (IRs) as well as predicted DnaA and IHF binding sites (mixed shapes). The sequences of the iteron-like DRs are presented next to the relevant diagrams with a consensus sequence shown for DRs of plasmids with related REP modules. Blue arrows indicate the *rep* genes and their transcriptional orientation. Specific motifs identified within the aa sequences of the Rep proteins are indicated by colored rounded bars. A+T and G+C indicate DNA regions of lower or higher than average G+C content, respectively. The components of the REP modules are not shown to scale.

Comparative sequence analysis revealed that the analyzed plasmids of *Paracoccus* spp. contain different types of replication systems. Based on aa sequence similarities of the predicted Rep proteins, the presence of conserved motifs and the *oriV* structures, the REP modules could be classified into four groups, some representing novel replication system types. The genetic structure of these REP modules is illustrated in Figure 2 and the nucleotide sequences of the predicted *oriV*s are presented in Additional file 2: Figure S1.

### REP modules of pAES2, pAES3, pHAE1, pHAE2, pMARC1, pMARC4 and pMOS7

The REP modules of plasmids pAES2, pAES3 (*P. aestuarii* DSM 19484), pHAE1, pHAE2 (*P. haeundaensis* LMG P-21903), pMARC1, pMARC4 (*P. marcusii* DSM 11574) and pMOS7 (*P. marcusii* OS22) encode replication initiation proteins (Rep) of the Rep_3 superfamily (PFAM: PF01051), which show significant level of aa sequence identity with RepB-type proteins from *Rhodobacterales*, whose phylogenetic analysis was presented by Petersen et al. [15]. Comparative analysis of the sequences and structures of these modules identified three distinct subgroups, present in the following plasmids: (i) pAES2, pAES3, pHAE1, pMARC4, (ii) pMARC1, pMOS7 and (iii) pHAE2. 

#### pAES2, pAES3, pHAE1 and pMARC4

The Rep proteins of the first subgroup show aa sequence identity ranging from 54 to 81% (highest identity observed between Reps of pHAE1 and pAES2). Database searches revealed that related proteins are commonly encoded by alphaproteobacterial plasmids. The best BLAST hits were obtained for replication initiator proteins of small plasmids: pMG160 of *Rhodobacter blasticus* (accession no NP_775696) [16] (70-78% aa sequence identity) and pWKS1 of *Paracoccus pantotrophus* DSM 11072 (accession no NP_775696) [12] (66-72% identity). 

The predicted *oriV*s of pAES2, pAES3, pHAE1 and pMARC4 were identified upstream of the *rep* genes (Figure 2). These highly conserved regions contain (i) three long iteron-like directly repeated sequences (DR1-DR3) of 19 bp, separated one from another by 2 bp spacers, and (ii) a single inverted partial iteron situated adjacent to the start codon of the *rep* gene (see Figure S1 in the supplemental material). As shown in Figure 2, the iterons of the analyzed plasmids are not identical, which suggests that these replicons belong to different *inc* groups. The highest level of sequence identity was observed between DRs of plasmids pHAE1 and pAES2, while DRs of pMARC4 were most divergent (Figure 2). A common feature of all distinguished iterons is the presence of a 3’-end G+C-rich sequence. Within the *oriV*-containing region of pMARC4 we also identified a palindromic sequence (5'-AGCCTTGCAAGGCT-3'), located upstream of the identified iterons. 

#### pMARC1 and pMOS7

Both REP modules are highly related and their Rep proteins share 96% aa sequence identity. Homologous replication initiator proteins are encoded by many plasmids residing in strains classified within the *Beta*- and *Gammaproteobacteria*. The highest level of identity (46%) was with the Rep of the small plasmid pHLHK19 of *Laribacter hongkongensis* HLHK19 (*Betaproteobacteria*) (accession no ABC70160). 

The predicted *oriV*s of pMOS7 and pMARC1 are placed upstream of the *rep* genes and in order they contain (i) three putative 16-bp iterons (DR1-DR3) separated by spacer sequences of 5 bp (Figure 2), (ii) a putative DnaA-box situated 2 bp downstream of DR3 (5’-TcATCCACA-3’ in pMARC1 and 5’-TTATCCACA-3’ in pMOS7; nucleotide not matching the consensus DnaA box, 5'-TTT/ATNCACA-3' [17], shown in lowercase), and (iii) 27-bp-long IR sequences (separated by 14 bp), which cover the predicted promoter, ribosome binding site (rbs) and ATG start codon of the *rep* gene (Figure S1). 

#### pHAE2

Plasmid pHAE2 also contains an iteron-type *oriV*, composed of (i) five 19-bp repeats (DR1-DR5), separated by spacer sequences of 3 bp, and (ii) identical IRs of 16 bp (separated by 22 bp), which precede the predicted iterons (Figure 2 and Figure S1). A BLAST search with the amino acid sequence of the pHAE2 Rep protein identified only two proteins with significant sequence similarity, encoded by *Sphingobium yanoikuyae* XLDN2-5 (accession no ZP_09907537) (60% aa sequence identity) and *Citreicella* sp. 357 (accession no ZP_10022073) (62% aa sequence identity) – both strains belonging to the *Alphaproteobacteria*. Weak homology to replication proteins of several *Acinetobacter* spp. plasmids (*Gammaproteobacteria*) was also observed. 

### REP modules of pAES1, pAES7, pMARC3 and pMOS2

 Another group of REP modules (present in plasmids pAES1, pAES7, pMARC3 and pMOS2) encode replication initiation proteins containing predicted helix-turn-helix (HTH) domains (HTH_36) conserved in many transcription regulators (PFAM: 13730). Based on aa sequence comparisons, these REP modules were divided into two subclasses comprising (i) pAES7, pMARC3, pMOS2, and (ii) pAES1. 

#### pAES7, pMARC3 and pMOS2

The Rep proteins of these three plasmids have aa sequence identities ranging from 57 to 62%. Homologous proteins are commonly encoded by many alphaproteobacterial strains, e.g. *Sphingobium xenophagum* QYY (accession no YP_195758), *Acetobacter pomorum* DM001 (accession no ZP_08242880) and *Acidiphilium cryptum* JF-5 (accession no YP_001220371) (approx. 60% aa sequence identity). 

These plasmids possess *oriVs* with different structures (Figure 2). However, all contain 2 iteron-like DRs (of 39 bp in pAES7, 18 bp in pMARC3 and 19 bp in pMOS2). The DRs of pMOS2 are overlapped by IRs of 14 bp (Figure S1). Interestingly, within the pAES7 *oriV*, an incomplete inverted iteron (14 bp) was identified, partially overlapping the start codon of the *rep* gene, which is analogous to the location of the partial iterons observed within the *oriV*s of pAES2, pAES3, pHAE1 and pMARC4. 

#### pAES1

The REP module of pAES1 is unique among *Alphaproteobacteria*, although related replication systems are common in enteric bacteria (*Gammaproteobacteria*). The pAES1-encoded Rep protein (Rep_pAES1_) shows highest aa sequence identity (46%) to the protein encoded by plasmid pKL1 of *Escherichia coli* KL4 (accession no NP_053155) (*Gammaproteobacteria*). The *oriV* of pKL1 is placed upstream of the *rep* gene and contains an IHF-box and several Rep binding sites [18]. The *oriV*-containing regions of pKL1 and pAES1 do not show significant nucleotide sequence similarity, but they contain analogously placed IR sequences: 16-bp long in pKL1 (perfectly matching repeats) and 28-bp long in pAES1 (5 mismatches) (Figure S1). 

Within the left and right IR of pAES1, two shorter (15 bp) directly repeated sequences were identified (DR1 and DR2), and a third copy of this repeat was detected elsewhere (DR3, located 70 bp downstream of DR2) (Figure 2 and Figure S1). 

### REP modules of plasmids pAES4 and pMARC2

The highly homologous REP modules of pAES4 and pMARC2 do not possess iteron-like DRs. Upstream of the *rep* genes they contain only long inverted repeats, covering the predicted *rep* promoters (Figure 2 and Figure S1). The IRs of pAES4 form a long (46 bp) imperfect (3 mismatches) palindromic sequence, while those of pMARC2 (18 bp) are identical and are separated by a 4-bp spacer sequence (Figure S1). 

The Rep proteins of these plasmids show 92% aa sequence identity. Both contain a predicted HTH motif typical of transcriptional regulators of the MarR family. Homologous Rep proteins are not common in other bacteria. Only four proteins with significant aa sequence identity (47-55%) (annotated as hypothetical proteins) were identified in the NCBI databases – all from strains belonging to the *Alphaproteobacteria*. These are encoded by (i-ii) plasmids pACMV6 and pACMV8 of *Acidiphilium multivorum* AIU301 (accession nos YP_004277313 and YP_004277317, respectively), (iii) plasmid pAPA01-060 of *Acetobacter pasteurianus* IFO 3283-01 (accession no YP_003189587) and (iv) *Rhodospirillum photometricum* DSM 122 (sequence contig; accession no YP_005418451). All the aforementioned proteins are annotated as hypothetical proteins and they do not show sequence similarity to any other plasmid encoded Rep proteins. Therefore, the results of the comparative analysis strongly suggest that the Rep proteins of pAES4 and pMARC2 may be considered as the archetypes of a novel group of plasmid replication initiators.

### REP module of pMOS6

The REP module of pMOS6 encodes a predicted Rep protein (Rep_pMOS6_), which is related to proteins encoded by strains belonging to the *Alpha*-, *Beta*- and *Gammaproteobacteria* as well as the CFB group of bacteria (genus *Bacteroides*). The majority of these proteins are annotated as conserved hypothetical proteins of unknown function. However, more detailed analysis revealed that all of them contain domains conserved in the plasmid replication proteins of the RepL family from *Firmicutes* (PFAM: PF05732). Closely related Rep protein sequences were identified within a few plasmids, i.e. pTINT02 of *Thiomonas intermedia* K12, pML of *Bartonella schoenbuchensis* m07a (accession no ENN90461) and pPsv48C of *Pseudomonas savastanoi*, and some chromosomes, e.g. *Bartonella vinsonii* (accession no YP_007462201). Interestingly, besides Rep_pMOS6_ homologs, the three aforementioned plasmids encode other proteins that may be involved in replication initiation (RepC in pML and a replication protein with a primase domain in pTINT02 and pPsv48C). Plasmid pMOS6 encodes only a single Rep protein, which is sufficient for initiation of replication, as proved by the construction of a pMOS6 minimal replicon (data not shown). 

The *oriV*-containing region of pMOS6, identified upstream of the *rep* gene, contains a sequence (5’-gAACcTCTGTCTTG-3’) with similarity to the IHF-box distinguished in *Paracoccus methylutens* DM12 plasmid pMTH4 [19] and two identical 18-bp iteron-like repeated sequences, separated by 3 bp (Figure 2 and Figure S1).

### Toxin-antitoxin modules

Toxin-antitoxin modules confer plasmid stabilization in a population by eliminating plasmid-less cells at the post segregational level. Such genetic modules are composed of two elements: (i) a toxin protein that binds a specific cellular target and (ii) an antitoxin (protein or antisense RNA), which counteracts the toxin. BLAST searches revealed that seven of the analyzed plasmids (pAES4, pAES7, pMARC1, pMARC3, pMARC4, pMOS6 and pMOS7) contain putative toxin-antitoxin modules representing five TA groups: (i) *relBE*/*parDE*, (ii) *tad*-*ata*, (iii) *higBA*, (iv) *mazEF* and (v) *toxBA*. 

Plasmids pAES3 and pAES7 (*P. aestuarii* DSM 19484) contain highly related TA modules (94% nucleotide sequence identity) of the RelBE/parDE superfamily [20], composed of two short overlapping ORFs (4 bp overlap). The first ORFs of the predicted operons (*orf6* of pAES3 and *orf3* of pAES7) encode putative proteins with significant similarity to a number of plasmid-encoded antitoxins, classified within a large family of transcriptional regulators containing a CopG/Arc/MetJ DNA-binding domain (cluster of orthologous groups COG3609). The highest similarity was to the TA module antitoxin of plasmid pAMI3 of *Paracoccus aminophilus* JCM 7686 [10] (78 and 79% aa sequence identity, respectively). The downstream ORFs (*orf5* of pAES3 and *orf4* of pAES7) encode putative proteins with substantial homology to toxins of the ParE family (COG3668), with the highest similarity to the toxin of the aforementioned pAMI3 TA module [10] (72 and 75% aa sequence identity, respectively).

Interestingly, plasmid pAES7 also carries another TA module (*orf14-orf15*) representing the *tad-ata* group, whose archetype was identified in plasmid pAMI2 of *Paracoccus aminophilus* JCM 7686 [21]. A related module is also present within plasmid pMOS6 of *P. marcusii* OS22 (*orf11-orf12*) (Figure 1). The Tad-related toxins are encoded by the first genes of the predicted TA operons. They belong to a large family of proteins (COG4679), exhibiting significant sequence similarity to the RelE toxins (*relBE*-type TA modules), which act as mRNA-cleaving RNAses [22]. Comparative sequence analysis of the antitoxins of pAES7 and pMOS6 (encoded downstream of the *tad*-homologs) revealed that these proteins belong to COG5606 and COG1396, respectively, and they contain a HTH motif typical of the Xre/Cro family.

An analogous genetic organization (toxin gene upstream of the antitoxin gene) was also observed in the TA system of pAES4 (*P. aestuarii* DSM 19484), classified within the *higBA* family [23]. This TA module is composed of two ORFs (*orf8* and *orf9*), separated by a 10-bp intergenic region, encoding proteins with the highest level of aa identity to the killer chromosomal protein of *Rhodopseudomonas palustris* CGA009 (accession no NP_947628) (68%) and the antitoxin of the Xre family encoded by another strain of *R. palustris* – BisB18 (accession no YP_534586) (70%), respectively. 

Plasmid pMARC4 of *P. marcusii* DSM 11574 carries two overlapping (4 bp) ORFs: *orf3* and *orf4*. The latter encodes a predicted toxin with substantial similarity (63% aa sequence identity) to the PemK-like protein of *Chlorobium phaeobacteroides* BS1 (*mazEF* family of TA systems). Interestingly, the *orf3*-encoded protein displays 65% identity to a hypothetical protein of plasmid pDSHI01 of *Dinoroseobacter shibae* DFL 12, and is quite different from typical MazE-type antitoxins. BLAST searches revealed that gene pairs homologous to *orf3*-*orf4* are conserved in many bacterial genomes (mainly in plasmids). Our analysis suggests that the TA hybrid module of pMARC4 might be considered the prototype of a new subgroup within the MazEF TA family. 

Plasmids pMARC1, pMARC3 (*P. marcusii* DSM 11574) and pMOS7 (*P. marcusii* OS22) contain related pairs of genes (*orf5*-*orf6*, *orf8*-*orf9*, *orf6*-*orf7*, respectively), which represent a novel group of TA modules, that we designate the *toxAB* family (Figure 1). Homologous loci were distinguished by Leplae et al. [24], but none of them were analyzed at the molecular level. The first genes of the predicted TA operons encode DUF497 proteins (predicted toxin; ToxB), while the genes in the second position were classified into the COG3680 (pMOS7 and pMARC1) or COG3514 (pMARC3) orthologous groups (putative antitoxins; ToxA). The predicted secondary structure of the COG3680 and COG3514 antitoxins is highly conserved. Proteins of both groups contain, in their C-terminal regions, a RHH_1 domain related to a domain of the CcdA antitoxin of the *ccdAB* TA system [25]. The most closely related TA module was identified within *Desulfomicrobium baculatum* DSM 4028 (accession nos YP_003157266 and YP_003157267). 

### Restriction-modification module

One of the analyzed plasmids (pAES2 of *P. aestuarii* DSM 19484) contains a putative type II restriction-modification (R-M) system (Figure 1). Similarly to TA, such systems may increase the stability of plasmids by killing plasmid-less cells [26]. The RM module of pAES2 is composed of overlapping *orf3* and *orf4* (1-bp overlap). The orf3 protein shares substantial similarity with a large number of proteins annotated as m5C methyltransferases (MTases) (PFAM: PF00145). The predicted pAES2 MTase contains six (I, IV, VI, VIII, IX and X) of the ten amino acid sequence motifs (placed in conserved order) characteristic of m5C MTases, including an invariant Pro-Cys dipeptide in the catalytic motif IV [27] (data not shown). The *orf4*-encoded protein is similar to restriction endonuclease NgoMIV, which recognizes the sequence 5’-GCCGGC-3’. The most closely related R-M module was identified in plasmid pAOVO02 of *Acidovorax* sp. JS42 (accession nos YP_974088 and YP_974088) with 89% and 93% aa sequence identity of the MTases and endonucleases, respectively.

### Partitioning module

Partitioning systems (PAR), which allow proper segregation of plasmid copies upon cell division, are components of the vast majority of large low copy number plasmids. Only one of the plasmids analyzed in this study (pMARC3 of *P. marcusii* DSM 11574; 10,672 bp) contains a predicted PAR module of typical structure, composed of two *par* genes (*orf2* and *orf3*) and a centromere-like partitioning site (Figure 1). 

BLAST searches revealed that the deduced aa sequence of Orf2 is similar over its entire length to a large number of partitioning ATPases (ParA), with highest identity (44%) to the putative ParA protein of plasmid pMRAD03 of the alphaproteobacterium *Methylobacterium radiotolerans* JCM 2831 (accession no YP_001776801). 

Detailed analysis of Orf2/ParA revealed the presence of a sequence motif [KGGSGKS] matching the canonical sequence [KGG(T/N/V)GKT] of a deviant Walker A motif, which is characteristic for ATPases of type I partitioning modules [28]. The *orf3*-encoded polypeptide of pMARC3 (putative ParB) displays only slight homology to a hypothetical protein of *Bacillus* sp. 2_A_57_CT2 (accession no ZP_08007698) (30% aa sequence identity), whose gene (as in pMARC3) is associated with that encoding a partitioning ATPase. 

The putative centromere-like site (*parS*) of the pMARC3 PAR module is located within the promoter region of *parA*, which consists of three non-identical 13-bp-long repeated sequences. 

Taking into account its structure (i.e. the presence of a small *parB* gene and the location of the *parS* site) and the results of detailed comparative analyses, the predicted PAR module of pMARC3 was classified into the Ib group of partitioning systems [28].

### Modules for mobilization for conjugal transfer

Many plasmids are capable of horizontal transfer by conjugation. According to their transfer ability, they may be grouped into two categories comprising self-transmissible and mobilizable replicons. The latter grouping contains MOB DNA regions, which carry genetic information essential for the processing of conjugative DNA. The MOBs are usually composed of two elements: an *origin* of transfer (*oriT*) and a gene coding for relaxase, which nicks DNA at the *oriT* sites. The transfer of mobilizable plasmids requires a membrane-associated mating pair formation complex, which may be provided by self-transmissible plasmids or integrative and conjugative elements (ICE) co-residing in the cell [29]. 

None of the 14 *Paracoccus* spp. plasmids was a self-transmissible replicon, but nine of them contained predicted MOB modules (Figure 1). Based on comparative analysis of the relaxase aa sequences, these proteins (and the MOB modules encoding them) were classified within the Mob_Q_, Mob_P_ or Mob_V_ families [30]. 

#### MOB_Q_ modules

The MOB_Q_ family constitutes a diverse group, comprising several subgroups (clades) [30]. The MOBs of paracoccal plasmids represent the MOB_Q1_ (pHAE1) and MOB_Q3_ (pAES7, pMARC3 and pMOS6) clades. The overall genetic organization of the MOB modules and the conserved sequence motifs of their relaxases are shown in Figure S2 in the supplemental material. 

BLAST searches revealed that plasmid pHAE1 (*P. haeundensis* LMG P-21903) encodes a protein with 34% aa sequence identity to the MobA relaxase of a broad-host-range (BHR) plasmid RSF1010 – the archetype of the MOB_Q_ family. A putative *origin* of transfer was identified upstream of the pHAE1 *mobA* gene (5’-AAAtaCATAAGTGCGCCCTCCC-3’), showing similarity to the MOB_Q_ family *oriT* consensus sequence (5’-NWACCNNTAAGTGCGCCCTYNN-3’) [31] (residues matching the consensus are shown in uppercase). Closely related MOB modules are encoded by several mobilizable plasmids, including pAB6 of *Neisseria meningitidis*, pP of *Salmonella enteritidis* and ColE2-P9 of *E. coli* [31]. 

The MOBs of pMARC3 (*P. marcusii* DSM 11574) and pMOS6 (*P. marcusii* OS22) (MOB_Q3_ clade; Additional file 3: Figure S2) are composed of two non-overlaping ORFs encoding, respectively, MobA relaxase and mobilization protein C (MobC), while the MOB of pAES7 is defective (it carries a truncated *mobA* gene, lacking its proximal part). The pMARC3 and pMOS6 relaxases exhibit the highest aa sequence identity (86%) to the MobA protein encoded by plasmid pAMI3 of *Paracoccus aminophilus* JCM 7686 [10]. The predicted *oriT*s of pMARC3 and pMOS6 were identified between divergently oriented *mobA* and *mobC* genes. These sequences are nearly identical (5’-ATAAGTGGGCACTTCGTGTCTTGCACCCTAt/c-3’; non-conserved nucleotides are shown in lowercase) and they show significant similarity to the putative *oriT* of pAMI3 [10]. Plasmid pAES7 does not contain related sequences. 

#### MOB_P_ modules

Two of the analyzed plasmids, pMARC2 (*P. marcusii* DSM 11574) and pAES4 (*P. aestuarii* DSM 19484), encode relaxases of the MOB_P_ family, classified within the MOB_P5_(MOB_HEN_) clade (Figure S2). MOB_P_ encodes the largest group of relaxases, which are closely related to those of the MOB_Q_ family. The prototype of the MOB_P5_(MOB_HEN_) relaxases is protein MbeA of plasmid ColE1 [30]. 

The MOB modules of pMARC2 and pAES4 are composed of three overlapping and convergently oriented ORFs: *mobA* (encoding the relaxase), *mobB* and *mobC* (Figure 1). The predicted MobA, MobB and MobC polypeptides exhibit significant similarity to the corresponding proteins encoded by plasmid pAsal2 (MOB_P5_ family) of *Aeromonas salmonicida* subsp. *salmonicida* [30]. The predicted *oriT* sequences of pMARC2 and pAES4 are placed upstream of the *mobC* gene and are highly conserved – they differ in only 3 nucleotides (5’-GGGGGATTGAAGGGGGCCAa/ca/ta/gGCCCCCTCACAAGC-3’; non-conserved nucleotides are shown in lowercase). A homologous DNA region (80% identity) is also conserved in plasmid pAsal2 (accession no AJ508383; nt position 590-621). 

#### MOB_V_ modules

Plasmids pMARC4 (*P. marcusii* DSM 11574), pMOS2 (*P. marcusii* OS22) and pAES1 (*P. aestuarii* DSM 19484) encode relaxases that are members of the MOB_V_ family (MOB_V2_ clade) (Figure S2). Most of the plasmids encoding MOB_V_ relaxases were identified in *Firmicutes* and *Bacteroidetes*, with the exception of the MOB_V2_ clade, which comprises plasmids of *Proteobacteria* and *Cyanobacteria* (pBBR1 of *Bordetella bronchiseptica* is an archetype of the group) [30]. Plasmids pMARC4, pMOS2 and pAES1 encode single mobilization proteins (MobA), which share the highest level of aa sequence identity (40 to 60%) with the MobA protein encoded by the small cryptic plasmid pWKS1 of *Paracoccus pantotrophus* DSM 11072 [12]. Significant aa sequence identity (from 30 to 40%) with the pBBR1 relaxase was also observed (Figure S2). In each case the *oriT* sites are situated upstream of the *mobA* genes. The predicted *oriT*s of pMOS2 (AATTTGGaCgcagGaCAAATTGTCTAGTaAGTgcACATttttCTcaaaT-3’) and pMARC4 are highly related (62% nucleotide sequence identity; nucleotides conserved in pMARC4 are shown in uppercase), while the *oriT* of pAES1 is more divergent (nucleotides conserved in all three *oriT*s are underlined). 

### Additional genetic load

Besides the REP, TA, PAR, R-M and MOB modules, the analyzed plasmids contain diverse accessory genetic information, including two insertion sequences (ISs) in pAES7 and pMARC3 (Figure 1). Plasmid pAES7 carries the functional element IS*Paes2* (IS*427* group, IS*5* family), which was identified previously by its transposition into the trap plasmid pMAT1 [5]. IS*Paes2* is bordered by dinucleotide (TA) direct repeats (DR), which represent the duplicated target site of transposition. 

The IS of pMARC3 is a novel element that we have designated as IS*Pmar4* (1343 bp). It contains 12-bp-long imperfect terminal IR sequences (5'-ATGGc/tCCGCCCC-3') and carries a single ORF (*orf5*), encoding a predicted protein with similarity to transposases of the IS*110* family (IS*1111* group). IS*Pmar4* is not flanked by DRs in the pMARC3 genome, which is a typical feature of members of the IS*110* family. 

An intriguing feature of all but one of the plasmids (pMARC3) is the presence of related ORFs (*orf2*), placed downstream of the predicted *rep* genes (Figure 1). These ORFs encode putative proteins (Orf2) with low, but significant similarity [at least 30% aa identity (E value 1e-04)] to entry exclusion-like proteins 1 (Exc1). All of these predicted proteins contain a conserved helix-turn-helix domain (HTH_17) in their N-terminal region (Figure S3 in the supplemental material). 

Entry exclusion (EEX) systems prevent the entry of exogenous plasmids into a host cell carrying an identical or related EEX system. It is thought that EEX is a specific feature of all conjugative plasmids [32]. The exclusion phenotype has been observed for mobilizable plasmid ColE1, whose *exc1* and *exc2* genes were predicted to encode the EEX system [33]. Although subsequent studies [34] excluded the possibility of *exc1* and *exc2* involvement in plasmid exclusion, homologous genes are still being annotated and described in the literature as entry exclusion components, e.g. [35]. 

The examined plasmids of *Paracoccus* spp. were found to contain only one of these genes – *exc1*. This is not a unique feature, since related “orphan” *exc1*-like genes have been identified in several other plasmids, including pKlebB-K17/80 of *Klebsiella pneumoniae* [36] and pMWHK1 of *Pedobacter cryoconitis* BG5 [35]. The conserved position of this gene, accompanying different REP modules, strongly suggests that the Exc1 proteins may play an important role in the biology of these plasmids. However, their specific function has yet to be determined. 

Downstream of the *exc1* genes in several plasmids (pAES3, pAES4, pHAE1, pHAE2, pMARC1, pMARC2, pMOS2 and pMOS7), putative plasmids islets (PI) were identified, i.e. horizontally-acquired DNA regions of lower than average GC content (Figure 1). The ORFs encoded within these PI (listed in Table 2) show similarity to genes (mainly of unknown function) conserved in the chromosomes of many bacteria. 

**Table 2:** Plasmid islets (PI) identified in *Paracoccus* spp. plasmids.

		**GC content (%)**			
**Plasmid**	**PI size (bp) (position)**	**PI**	**Remaining part of the plasmid**	**ORF (aa)**	**Hypothetical function**
pAES3	2273 (1509-3781)	42.3	58.5	*orf3* (374)	Unknown
				*orf4* (93)	Unknown
pAES4	2218 (1303-3521)	52.3	62.4		
pHAE1	1142 (1400-2542)	47.7	61.8	*orf3* (87)	Unknown
pHAE2	2695 (1365-4059)	46.5	59.8	*orf3* (151)	Unknown
				*orf4* (473)	Unknown
pMARC1	1831 (1312-3143)	35.7	55.1	*orf3* (131)	Unknown
				*orf4* (389)	GTP pyrophosphokinase (RelA/SpoT domain-containing protein)
pMARC2	2218 (1303-3521)	39.5	57.7	*orf3* (415)	ATPase (SMC domain-containing protein)
				*orf4* (211)	Unknown
pMOS2	1949 (1042-2991)	43.4	58.6	*orf3* (113)	Unknown
pMOS7	2425 (1329-3754)	38.5	57.8	*orf3* (113)	Unknown
				*orf4* (314)	Reverse transcriptase (retron-like)

**Figure 3:**
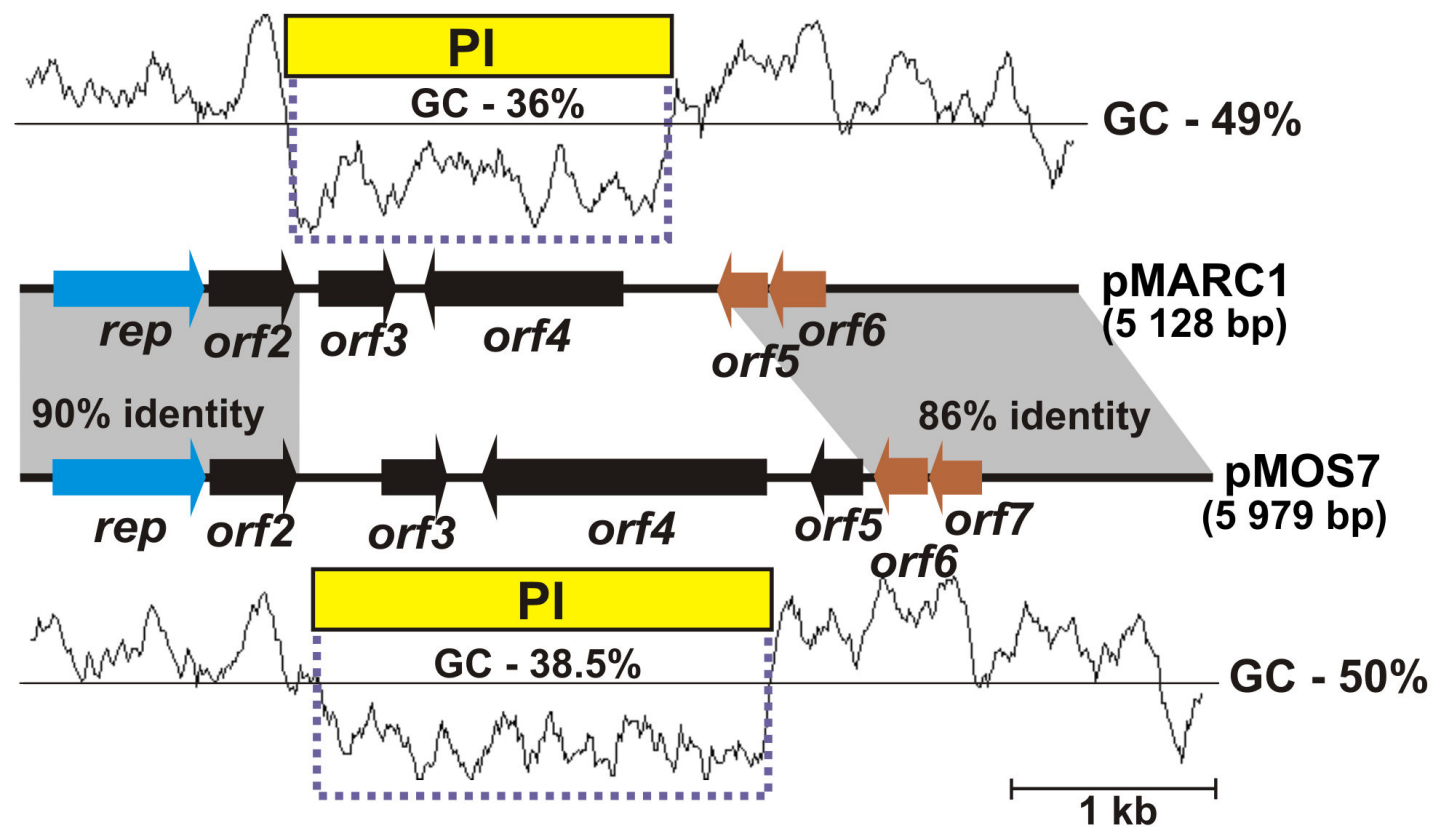
Comparison of the structure and G+C sequence profile of *P. marcusii* plasmids pMARC1 and pMOS7. Arrows show the transcriptional orientation of the genes and the color code indicates their predicted functions (as shown in Figure 1). Shaded areas connect homologous DNA regions. The PI regions (with indicated G+C content) are marked by yellow rectangles and dashed lines. The plot shows the G+C content of pMARC1 and pMOS7 sequences (the average values are given to the right).

The significant role of PI in shaping plasmid genomes was revealed by comparative analysis of the related plasmids pMARC1 and pMOS7 of *P. marcusii* (strains DSM 11574 and OS22, respectively). These plasmids have highly conserved backbones (REP and TA modules showing approx. 86% nucleotide sequence identity), but they contain different PIs (Figure 3). Plasmids pMARC2 and pHAE4 represent an analogous pair of related replicons containing different PIs (Figure 1). The PI of pMOS7 carries two ORFs, including *orf4* encoding a predicted retron-type reverse transcriptase, while that of pMARC1 is composed of *orf3*, encoding a putative DUF805 transmembrane protein and *orf4*, encoding a protein containing a conserved domain of RelA and SpoT proteins (both proteins are involved in the metabolism of the regulatory compound guanosine 3',5'-bis-pyrophosphate, ppGpp, which plays a crucial role in the bacterial stringent response). 

The PI of pMARC2, another plasmid of *P. marcusii* (strain DSM 11574), contains a pair of overlapping genes (1-bp overlap), *orf3* and *orf4*, conserved (in synteny) in several bacterial chromosomes. The first gene of this predicted module encodes a putative ATPase with significant similarity to the SMC proteins, which play an important role in chromosome condensation, packaging, partitioning and DNA repair [37]. The downstream *orf4* encodes a hypothetical protein of unknown function. The closest homologs of *orf3* and *orf4* were identified within the chromosome of *Pedobacter heparinus* DSM 2366 (accession nos YP_003093112 and YP_003093113, respectively). 

The largest of the plasmids analyzed in this study, pMARC4, does not contain a PI, but it does encode proteins possibly involved in carbohydrate metabolism. The predicted proteins show significant similarity to (i-ii) acyltransferases (Orf5 and Orf7), (iii) polysaccharide biosynthesis protein (Orf10), (iv) phosphoribosyltransferase (Orf11), (v) dolichyl-phosphate mannose synthase (Orf12), and (vi) glycosyltransferase (Orf13). Two other ORFs (*orf6* and *orf14*) encode a putative undecaprenyl-diphosphate phosphatase and UDP-glucose 6-dehydrogenase (EC 1.1.1.22), respectively. It has been demonstrated that Orf6 homologs are involved in the synthesis and recycling of undecaprenyl phosphate (Und-P), a lipid carrier of glycan biosynthetic intermediates of carbohydrate polymers exported to the bacterial cell envelope [38], while Orf14 relatives are responsible for the NAD-dependent oxidation of UDP-glucose to UDP-glucuronic acid, a key component in the biosynthesis of gellan (extracellular polysaccharide of biotechnological value) [39]. Based on these similarities, it is likely that the pMARC4-encoded gene cluster may be involved in the biosynthesis of envelope-associated polysaccharides. 

Another plasmid, pMOS6 of *P. marcusii* OS22, besides several ORFs of unknown function, carries *orf4* encoding a putative zinc-dependent alcohol dehydrogenase (ADH_ZINC), containing a conserved signature sequence: G-H-E-x(2)-G-x(5)-[GA]-x(2)-[IVSAC] (where x indicates any amino acid; H is a zinc ligand) [40]. Related enzymes catalyze the oxidation of alcohols, with the concomitant reduction of nicotinamide adenine dinucleotide (NAD) [41]. The most similar alcohol dehydrogenase (99% aa sequence identity) is encoded by *Methylobacterium chloromethanicum* CM4 (accession no YP_002424246). 

### Distribution of related plasmids in genus *Paracoccus*

We analyzed the distribution of the replication modules of the pAES, pHAE, pMARC and pMOS plasmids in the genomes of 20 strains representing 19 species of *Paracoccus* spp. To do this, a specific DNA probe for each plasmid (*rep* gene fragment amplified by PCR and DIG-labeled) was used in dot blot hybridization to screen total DNAs isolated from the paracoccal strains. 

**Figure 4:**
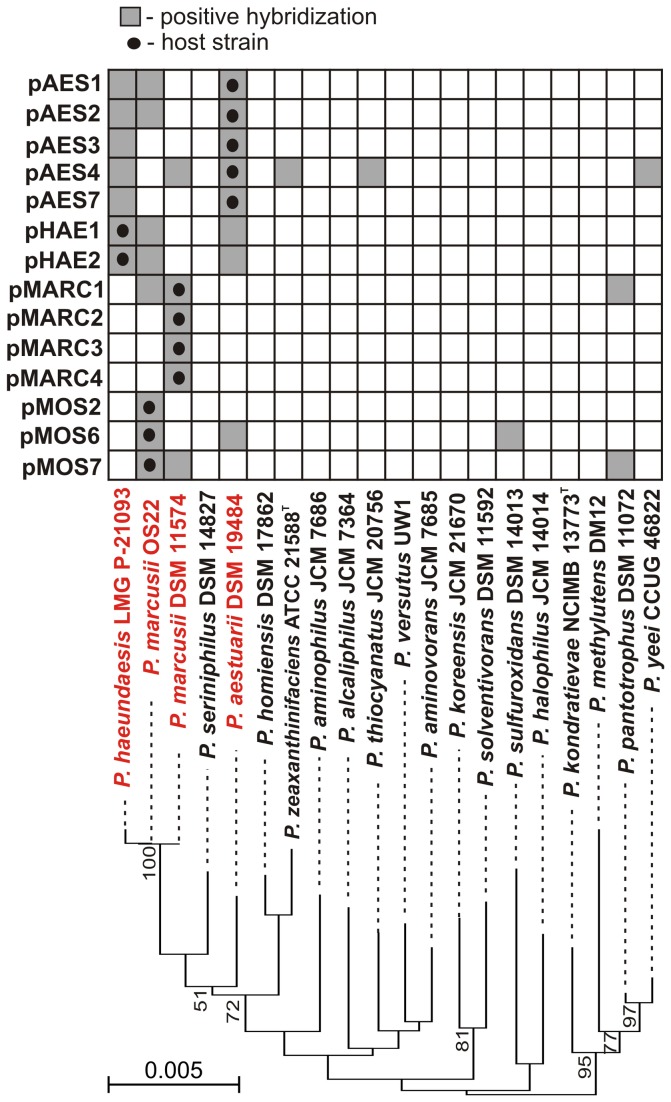
Distribution of the REP modules analyzed in this study in the *Paracoccus* spp. genomes. A specific DNA probe (fragment of a *rep* gene amplified by PCR and DIG-labeled) was prepared for each analyzed REP module and used in dot blot hybridization analysis with total DNA isolated from 20 strains of *Paracoccus* spp. The results are presented as a matrix. The relatedness of the tested *Paracoccus* strains is shown beneath by a phylogenetic tree based on their 16S rDNA sequences. The tree was constructed by the neighbor-joining algorithm with Kimura corrected distances. The statistical support for the internal nodes was determined by 1000 bootstrap replicates and values of >50% are shown. The *Paracoccus* strains from which the plasmids were isolated are denoted by red text.

This analysis revealed that the majority of the analyzed plasmids occur exclusively in the multireplicon carotenoid-producing species (LMG P-21093, OS22, DSM 11574, DSM 19484), although none of the replicons was present in all four strains (Figure 4). The strains *P. haeundaensis* LMG P-21093 and *P. aestuarii* DSM 19484 displayed an almost identical hybridization pattern (7 common replicons), which was also similar to that of *P. marcusii* OS22 (4 common plasmids). In contrast, the hybridization pattern of two strains of *P. marcusii* (OS22 and DSM 11574) was significantly different, indicating the presence of four strain-specific replicons (pMARC2, pMARC3, pMARC4 and pMOS2), which were unique among *Paracoccus* spp. (Figure 4).

A few related plasmids were also detected in other species, located on distant branches of the phylogenetic tree, which points to the role of horizontal gene transfer in the dissemination of these replicons. The most ubiquitous replicons were plasmids related to pAES4, which were detected in 6 strains, including *P. zeaxanthinifaciens* ATCC 21588T, *P. thiocyanatus* JCM 20756 and *P. yeei* CCUG 46822 (Figure 4). 

### Host range of the *Paracoccus* spp. plasmids

The host range of the paracoccal plasmids in several bacterial strains belonging to the *Alpha*-, *Beta*- or *Gammaproteobacteria* was examined. For this analysis we employed REP regions representing different groups of plasmids (Figure 2): (i-ii) pAES1 and pMOS7, (iii) pAES7, (iv) pMARC2 and (v) pMOS6. The REPs were cloned within the multiple cloning site (MCS) of the mobilizable vector pABW1 (Km^r^, *oriV* ColE1, *oriT* RK2) (see Methods for details) and the resulting plasmids were introduced into 9 strains of *Alphaproteobacteria* (Rif^r^ derivatives of *Paracoccus* spp. strains *P. alcaliphilus* JCM 7364, *P. aminophilus* JCM 7686, *P. aminovorans* JCM 7685, *P. kondratievae* NCIMB 13773, *P. pantotrophus* DSM 11072, *P. versutus* UW225, as well as *Rhizobium etli* CE3 and *Ochrobactrum* sp. LM19R, one strain of *Betaproteobacteria* (*Alcaligenes* sp. LM16R) and two strains of *Gammaproteobacteria* (*Pseudomonas* sp. LM7R and *E. coli* BR825

The constructed shuttle plasmids contained an *E. coli*-specific pMB1 (ColE1-type) replication system, which is functional in neither *Alpha*- and *Betaproteobacteria* nor in *Pseudomonas* sp. LM7R and *E. coli* BR825 (*Gammaproteobacteria*; the BR825 strain carries a mutation within the DNA polymerase I gene that blocks pMB1 replication). Therefore, the functions required for replication and maintenance of these plasmids in the tested hosts have to be provided by the paracoccal REP modules. It is important to mention that the *Paracoccus* spp. strains, in which the plasmids were tested for their ability to replicate, were not the original hosts of any of these plasmids, and most of these strains did not render a positive hybridization with the REP probes of such plasmids (Figure 4).

All of the shuttle plasmids were found to replicate exclusively in strains of *Alphaproteobacteria*, which indicates the relatively narrow host range of the tested REP modules. This is in agreement with the results of our previous studies, which showed that the plasmids of *Paracoccus* spp. are not promiscuous (e.g. [10]). 

 Members of the genus *Paracoccus* are not naturally competent for transformation, therefore conjugal transfer is the only efficient way of introducing of foreign DNA into their cells. Unfortunately, conjugative plasmids (or ICE elements) have not yet been identified in these bacteria. We also do not know whether such replicons are present in the natural host strains of the plasmids analyzed in this study (*P. aestuarii*, *P. haeundensis* and *P. marcusii*). However all of them (as well as many other *Paracoccus* spp. and numerous *Alphaproteobacteria*) carry mega-sized replicons, some of which could be self-transmissible. 

Until now, the complete nucleotide sequences of only three *Paracoccus* spp. genomes (with defined physical maps of chromosomes and plasmids) have been deposited in the NCBI database (*P. denitrificans* PD1222, *P. aminophilus* JCM 7686 and *Paracoccus* sp. N5). Our detailed *in silico* analysis revealed that each of the strains carries one replicon (chromosome II, megaplasmid pAMI8 and a replicon referred to as contig 3, respectively) containing a complete predicted type IV secretion system, with a potential to support the conjugal transfer of mobilizable plasmids. Plasmid mobilization might be therefore a frequent phenomenon in *Paracoccus* spp., especially when the fact that the majority of the plasmids characterized in this study (9 replicons) contained the MOB modules is taken into account.

### Vector cassette construction

To facilitate genetic manipulation of the carotenoid-producing strains of *Paracoccus* spp. two vector cassettes were constructed (see Methods for details). We used REP regions of plasmids pMOS6 (*P. marcusii* OS22) and pAES7 (*P. aestuarii* DSM 19484), which were free of the majority of restriction sites commonly found in the MCSs of many cloning vectors. The construction of these cassettes was performed according to the general scheme used for the generation of the DIY (Do It Yourself) cassettes, described in our previous study [10]. 

**Figure 5:**
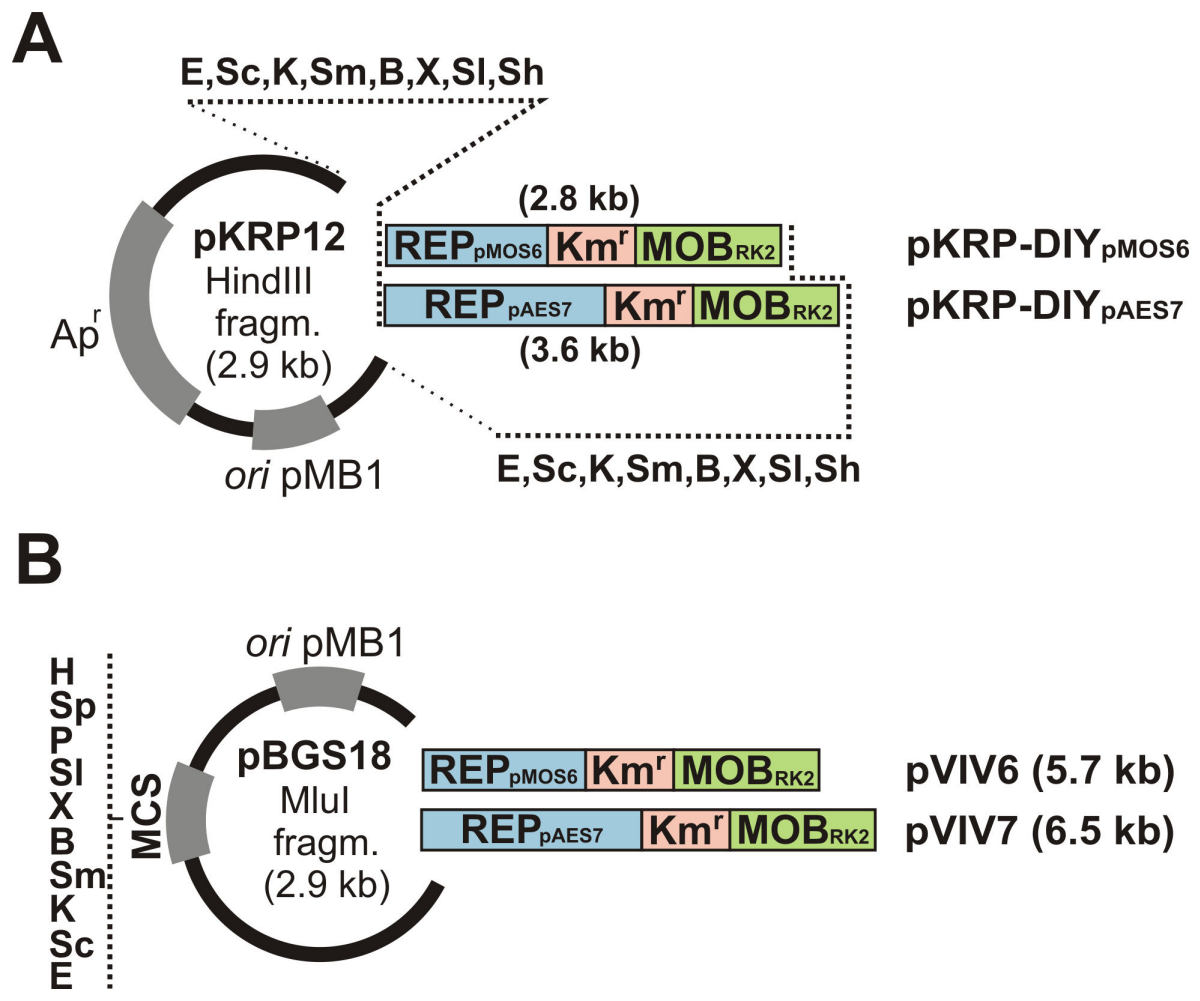
The plasmids containing the DIY cassettes constructed in this study. A. pKRP-DIY plasmids. B. pVIV mobilizable shuttle vectors. The plasmids contain DIY_AES7_ and DIY_MOS6_ cassettes composed of REP modules (of plasmids pAES7 and pMOS6, respectively), a Km^r^ gene and a MOB module derived from BHR plasmid RK2. B – BamHI, E – EcoRI, H – HindIII, K – KpnI, P – PstI, Sc – SacI, Sh – SphI, Sl – SalI, Sm – SmaI, X – XbaI.

The cassettes contain (i) the REP regions, (ii) a kanamycin resistance gene, providing a selectable marker convenient for *Paracoccus* spp. (and other *Alphaproteobacteria*), and (iii) a MOB module, which enables conjugal transfer of the plasmids in the presence of a helper, functional transfer system (the MOB module originated from BHR conjugative plasmid RK2, commonly used in the construction of vectors for gram-negative bacteria). Both cassettes, designated DIY_pMOS6_ and DIY_pAES7_, also contain polylinkers with a number of restriction sites to facilitate their insertion into different locations (plasmids pKRP-DYI_pMOS6_ and pKRP-DIY_pAES7_; Figure 5). The pKRP-DIY plasmids are not cloning vectors and they serve exclusively as a source of the DIY cassettes. Insertion of a single cassette into any *E. coli* plasmid can create a mobilizable shuttle vector. 

The cassettes were used to construct two mobilizable *E. coli*-*Paracoccus* spp. shuttle vectors pVIV6 and pVIV7, whose genetic organization is shown in Figure 5. These vectors also carry a MluI restriction fragment of *E. coli*-specific plasmid pBGS18 containing (i) a replication system originating from pMB1 (non functional in *Paracoccus* spp.) and (ii) a selection cassette (MCS and the *lacZ’* gene) enabling the identification of recombinant molecules by "blue-white" screening. These shuttle vectors proved to be good cloning vectors with specific features determined by the DIY cassettes (data not shown). 

There are also available some other cloning vectors based on the REP modules of *Paracoccus* spp. plasmids. They contain replication systems of pTAV1 (*P. versutus* UW1) [42] or pAMI plasmids of *P. aminophilus* JCM 7686 [10]. Although all of them are functional in *Alphaproteobacteria*, their stability significantly varies in different hosts [10]. Therefore there is a need to enrich the pool of such vectors, which will enable selection of the most convenient one for a given host and task. 

## Conclusions

The findings of this study provide a molecular insight into the genomes of a pool of small plasmids occurring in four carotenoid-producing strains of the genus *Paracoccus* (*Alphaproteobacteria*). Three of these strains (*P. haeundaensis* LG P-21903, *P. marcusii* OS22 and patented *P. marcusii* DSM 11574) synthesize astaxantin, which is a commercially produced carotenoid used in a range of industrial applications. The DIY cassettes and shuttle vectors constructed in this study may facilitate further genetic analysis of these biotechnologically important bacteria. 

Until now, small alphaproteobacterial plasmids have been neglected by researchers, e.g. complete nucleotide sequences of only four such plasmids of *Paracoccus* spp. were available - pWKS1 of *P. pantotrophus* DSM 11072 [12] and pAMI2, pAMI3, pAMI7 of *P. aminophilus* JCM 7686 [10,11]. Interest has almost exclusively been focused on mega-sized replicons, which appear to be less diverse than the plasmids characterized in this study. Comparative analysis has revealed that the plasticity and diversity of *Paracoccus* spp. plasmids result from (i) recombinational shuffling of genetic modules of the plasmid backbones, (ii) insertion of exogenous foreign DNA, including commonly identified plasmid islets (PIs), as well as (iii) the acquisition of novel replicons. Our detailed description of the genetic content of these plasmids allows prediction of the possible origin of individual genes (or sets of genes) and the direction of horizontal gene flow in this group of bacteria.

Some of the plasmids characterized in this study (pAES1, pAES2, pAES3, pEAS4, pHAE1 and pHAE2).carry replication systems which occur exclusively in the phylogenetically closely related orange-pigmented strains of *Paracoccus* spp. (Figure 4). This suggests that their ancestor replicons might have been acquired a long time ago (from an evolutionary point of view) by a progenitor strain. In contrast, a few plasmids (pMARC2, pMARC3, pMARC4, pMOS2), present only in single strains, are unique among *Paracoccus* spp., which suggests their relatively recent acquisition. Interestingly, two of the aforementioned replicons (pAES4 and pMARC2) encode related Rep proteins, which may be considered as archetypes of a novel group of plasmid replication initiators.

Analysis of plasmid host range strongly suggests that the *Alphaproteobacteria* is a kind of “isolated island”, since all plasmids identified so far in these bacteria (including those analyzed in this study) are narrow-host-range class-specific replicons. They do not replicate in *Beta*- or *Gammaproteobacteria*, and *vice versa*, foreign plasmids (with the exception of broad host range replicons) do not replicate in alphaproteobacterial hosts. This isolation seems to be the main factor limiting plasmid diversity in *Alphaproteobacteria*. These observations also suggest that *Alphaproteobacteria* may encode as yet unidentified host-specific factors that are crucial for the maintenance of certain types of plasmids. The identification of such factors is an immediate goal of our future studies. 

## Materials and Methods

### Bacterial strains and culture conditions

The following strains of the genus *Paracoccus* were used in this study: (i) *P. aestuarii* [43], (ii) *P. haeundaensis* LG P-21903 [44], (iii) *P. marcusii* [45], (iv) *P. marcusii* OS22 [46] as well as (v-xviii) *P. alcaliphilus* JCM 7364 [47], *P. aminophilus* JCM 7686 [48], *P. aminovorans* JCM 7685 [48], *P. halophilus* JCM 14014 [49], *P. homiensis* DSM 17862 [50], *P. kondratievae* NCIMB 13773 [51], *P. koreensis* JCM 21670 [52], *P. methylutens* DM12 [53], *P. pantotrophus* DSM 11072 [54], *P. pantotrophus* KL100 [6], *P. seriniphilus* DSM 14827 [55], *P. solventivorans* DSM 11592 [56], *P. sulfuroxidans* DSM 14013 [57], *P. thiocyanatus* JCM 7364 [58], *P. versutus* UW1 [59], *P. versutus* UW225 [60], *P. yeei* CCUG 46822 [61] and *P. zeaxanthinifaciens* ATCC 21588 [62]. Other strains used in this study were (i) *Rhizobium etli* CE3 [63], *Ochrobactrum* sp. LM19R [10], (*Alphaproteobacteria*) (ii) *Alcaligenes* sp. LM16R [10] (*Betaproteobacteria*), and (iii) *Eschericha coli* – strains TG1 [64], BR825 [65] and *Pseudomonas* sp. LM7R [66] (*Gammaproteobacteria*). As recipients in triparental matings, rifampicin-resistant (Rif^r^) derivatives of the wild-type strains were used. Bacteria were grown in LB (Luria Bertani) medium [67], at 37°C (*E. coli*) or 30°C (other strains). *P. homiensis* DSM 17862 was cultivated in Marine Broth (Difco) and *R. etli* CE3 in TY medium [68]. Where necessary, the medium was supplemented with kanamycin (50 μg/ml), streptomycin (50 μg/ml) and rifampicin (50 μg/ml).

### Plasmids used and constructed in this study

The following plasmid vectors were used: (i) pABW1 (Km^r^; *ori* pMB1; *oriT* RK2; MCS-*lacZ*') [69], pBGS18 (Km^r^; *ori* pMB1; MCS) [70], pBluescript KSII (Ap^r^; *ori* pMB1; MCS-*lacZ*') [71], pGEM-T Easy (Promega), pKRP12 (Ap^r^; Km^r^; *ori* pMB1) [72], pDIY-KM (Ap^r^; Km^r^; *ori* pMB1) [10]. Plasmids constructed in this study were (i-v) pABW-AES1, pABW-AES7, pABW-MARC2, pABW-MOS6 and pABW-MOS7 – shuttle plasmids used for host range analysis, (vi-ix) pKRP-DIY_AES7_, pKRP-DIY_MOS6_, pBS-DIY_AES7_ and pBS-DIY_MOS6_ – containing DIY cassettes, and (x-xi) shuttle vectors pVIV6 and pVIV7. 

Shuttle plasmids pABW-AES1, pABW-AES7, pABW-MARC2, pABW-MOS6 and pABW-MOS7 (containing REP regions of plasmids pAES1, pAES7, pMARC2, pMOS6 and pMOS7, respectively) were constructed by cloning of REP-containing plasmid restriction fragments (pAES7, pMARC2, pMOS6 and pMOS7) or DNA fragment amplified by PCR (pAES1; primers used are listed in Table S2 in the supplemental material) into the MCS of mobilizable vector pABW1. 

The plasmids representing the source of the DIY cassettes were constructed in several steps. First, the REP modules of pAES7 and pMOS6 were amplified by PCR (pMOS6; primers listed in Table S2) or recovered within 1.9 kb SacI-NotI restriction fragment (pAES7) and cloned into vector pGEM-T Easy. These modules were then excised from the recombinant plasmids with restriction endonuclease NotI and cloned into compatible sites of plasmid pBS-MOBKm (pBluescript SKII containing the Km^r^ cassette from pMBS1 and the BHR plasmid RK2 MOB module within its MCS). The REP-Km^r^-MOB cassettes were excised from the recombinant plasmids and inserted (i) between HindIII sites of plasmid pKRP12 (HindIII digestion of pKRP12 removed the original resistance gene cassette of this plasmid), yielding plasmids pKRP-DIY_AES7_ and pKRP-DIY_MOS6_, or (ii) cloned into the MCS of Bluescript SKII, yielding plasmids pBS-DIY_AES7_ and pBS-DIY_MOS6_. 

The mobilizable *E. coli*-*Paracoccus* spp. shuttle plasmids pVIV6 and pVIV7 were constructed by ligation of the DIY_pAES7_ or DIY_pMOS6_ cassettes (excised from pBS-DIY_AES7_ and pBS-DIY_MOS6_, respectively) with MluI-cleaved pBGS18. 

### Plasmid DNA isolation, genetic manipulations and PCR conditions

Plasmid DNA was isolated using a standard alkaline lysis procedure [73] and when required, purified by CsCl-ethidium bromide density gradient centrifugation. Total DNA was isolated from *Paracoccus* spp. using the procedure described by Chen and Kuo [74]. Southern hybridization analysis and common DNA manipulation methods were performed according to Sambrook and Russell [67]. Oligonucleotides used to generate molecular probes are listed in Table S2. PCR was performed in a Mastercycler (Eppendorf) using HiFi polymerase (Qiagen; with supplied buffer), dNTP mixture and total DNA of *Paracoccus* spp. with appropriate primer pairs (listed in Table S2). 

### Introduction of plasmid DNA into bacterial cells and plasmid stability assay

DNA was introduced into Rif^r^ (*Alcaligenes* sp. LM16R, *Ochrobactrum* sp. LM19R, *Paracoccus* spp. and *Pseudomonas* sp. LM7R) or Str^r^ (*R. etli* CE3) recipient strains by triparental mating as previously described [42]. Chemical transformation of *E. coli* cells was performed according to the method of Kushner [75]. The stability of plasmids was tested during growth under non-selective conditions. Stationary-phase cultures of plasmid-containing strains were diluted in fresh medium without antibiotic selection and cultivated for approximately 10, 20 and 30 generations. Samples were diluted and plated onto solid medium lacking selective antibiotics. One hundred colonies were tested for the presence of the Km^r^ marker by replica plating. Plasmid retention was determined from the percentage of kanamycin-resistant colonies.

### DNA sequencing

The nucleotide sequences of plasmids pMARC and pMOS were determined in the DNA Sequencing and Oligonucleotide Synthesis Laboratory at the Institute of Biochemistry and Biophysics, Polish Academy of Sciences, using a dye terminator sequencing kit and an automated sequencer (ABI 377 Perkin Elmer). Primer walking was used to complete the sequences. In the case of pMARC2, pMARC3 and pMOS2, unidirectional nested deletions within the cloned plasmid restriction fragments were generated by the use of exonuclease III and S1 nuclease (ExoIII/S1 deletion kit; MBI Fermentas). The nucleotide sequences of plasmids pAES and pHAE were determined by pyrosequencing performed by Genomed. 

### Bioinformatic analysis

Plasmid nucleotide sequences were analyzed using Clone Manager (Sci-Ed8) and Artemis software [76]. Similarity searches were performed using the BLAST programs [77] provided by the National Center for Biotechnology Information (NCBI) (http://blast.ncbi.nlm.nih.gov/Blast.cgi). Comparison searches of insertion sequences were performed with ISfinder [78]. Protein homology detection and structure prediction were performed using the HHpred program [79]. Protein families were identified using the PFAM database [80]. Helix-turn-helix motifs were predicted using the HELIX-TURN-HELIX MOTIF PREDICTION program [81]. Phylogenetic analyses were performed using the Phylogeny Inference Package – PHYLIP v3.69 [82], applying the neighbor-joining algorithm with Kimura corrected distances and 1000 bootstrap replicates. DNA sequence alignments obtained using ClustalW [83] were manually refined using the T-Coffee Multiple Sequence Alignment program [84]. The tree was rendered with TreeView version 1.6.6 [85].

### Nucleotide sequence accession numbers

Plasmid nucleotide sequences have been annotated and deposited in the GenBank database (accession numbers are given in parenthesis): pAES1 (JQ041633), pAES2 (JQ065021), pAES3 (JQ066766), pAES4 (JQ684025), pAES7 (JQ796370), pHAE1 (JQ066767), pHAE2 (JQ684024), pMARC1 (KC542384), pMARC2 (KC561053), pMARC3 (KC561054), pMARC4 (KC561055), pMOS2 (JQ664550), pMOS6 (JQ678602) and pMOS7 (JQ684023). 

## Supporting Information

Figure S1 **Nucleotide sequence of DNA regions containing the predicted origin of replication of the *Paracoccus* spp. plasmids analyzed in this study.** Iterons (DRs) are shown against orange background, while DnaA-boxes and IHF-box have violet and blue backgrounds, respectively. Inverted, repeated sequences are indicated by blue arrows. Predicted -35 and -10 promoter sequences are indicated by black frame.
(TIF)

Figure S2 **Comparison of sequence motifs identified in relaxases encoded within MOB modules of the *Paracoccus* spp. plasmids analyzed in this study.** The conserved motifs identified within the relaxase (MobA) proteins of analyzed plasmids were present in a form of alignments. Conserved amino acids, characteristic for each motif (according to Francia et al. [31]; Garcillan-Barcia et al. [30]), were shown against the blue background. Other conserved amino acids common in more than 50% of analyzed sequences are shown against black background, and those common in less than 50% have gray background. For the alignments additional MobA sequences of various mobilization plasmids, classified into appropriate category (according Francia et al. [31]; Garcillan-Barcia et al. [30]) were used.
(TIF)

Figure S3 **Multiple alignment of amino acid sequences of Exc1-like proteins encoded by *Paracoccus* spp. plasmids analyzed in this study.** For the alignment the Exc1-like proteins of the following plasmids were used: pMARC1, pMARC2, pMARC4 of *P. marcusii* DSM 11574, pMOS2, pMOS4, pMOS7 of *P. marcusii* OS22, pAES2, pAES3, pAES4, pAES7 of *P. aestuarii* DSM 19484, pHAE1, pHAE2 of *P. heaundaensis* LMG P-21903, pAMI3 of *P. aminophilus* JCM 7686 (YP_003305342), pSX-Qyy of *Sphingobium xenophagum* QYY (sequence distinguished in this work), pYAN-1 of *Sphingobium yanoikuyae* JCM 7371 (sequence distinguished in this work), pUT2 of *Sphingobium japonicum* UT26S (YP_003550321), as well as protein sequence annotated within a contig of an unfinished genomic project of *Sulfitobacter* sp. NAS-14.1 (ZP_00964870). Amino acids identical in at least 50% of the analyzed sequences are shown against a black background, while those common to at least 15% of the analyzed sequences have a gray background. The HTH motifs were distinguished by blue frame.
(TIF)

Table S1 **ORFs located within the *Paracoccus* spp. plasmids analyzed in this study.**
(DOC)

Table S2 **Oligonucleotide primers used in this study.**
(DOCX)
