# Supplementary figures and images for: Plasmids of Carotenoid-Producing Paracoccus spp. (Alphaproteobacteria) - Structure, Diversity and Evolution
Source: PLoS One. 2013 Nov 8;8(11):e80258. doi: 10.1371/journal.pone.0080258 (PMC3832669; doi:10.1371/journal.pone.0080258)

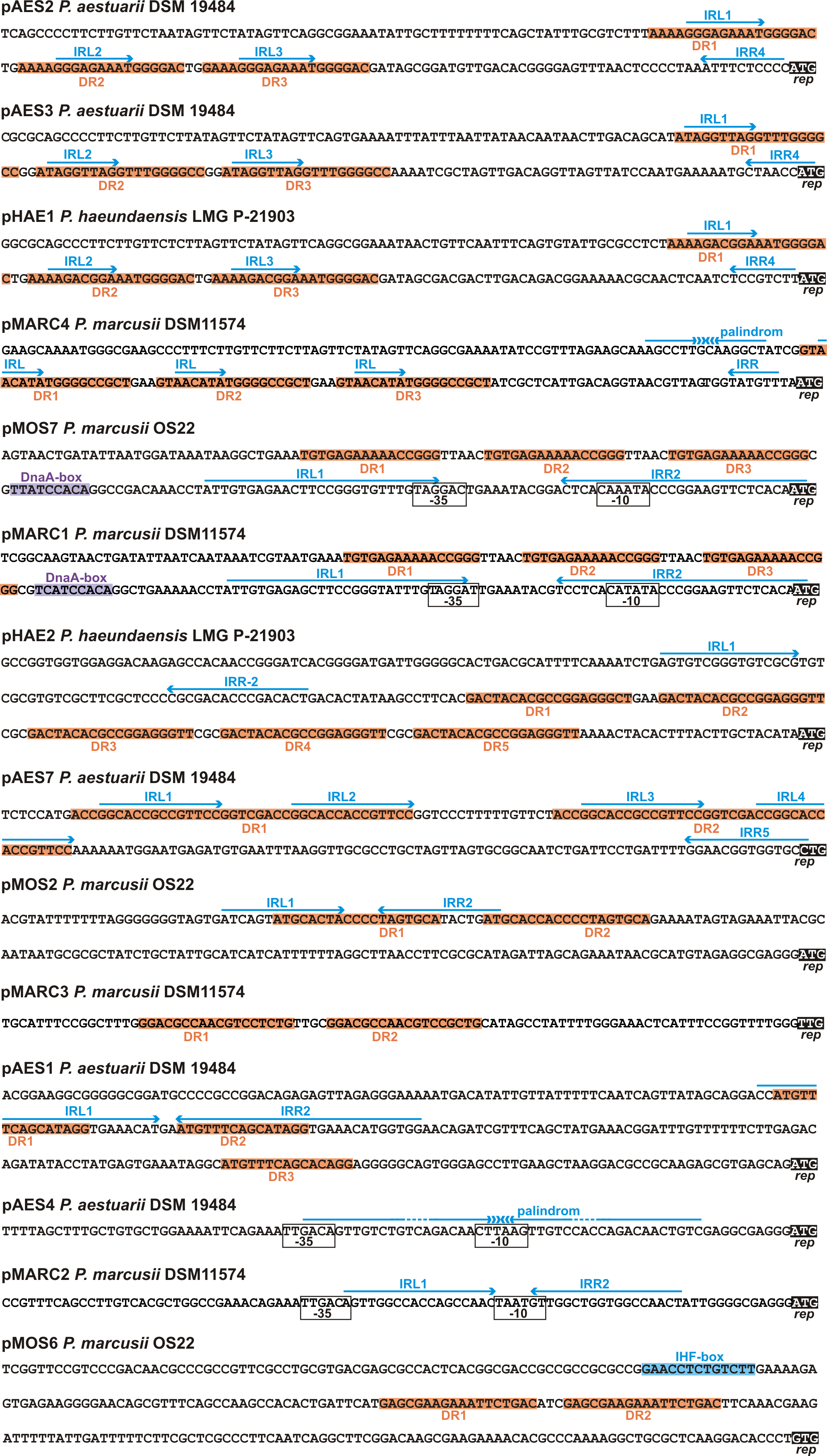

Supplement: Figure S1 — Nucleotide sequence of DNA regions containing the predicted origin of replication of the Paracoccus spp. plasmids analyzed in this study. Iterons (DRs) are shown against orange background, while DnaA-boxes and IHF-box have violet and blue backgrounds, respectively. Inverted, repeated sequences are indicated by blue arrows. Predicted -35 and -10 promoter sequences are indicated by black frame. (TIF) [file pone.0080258.s001.tif]

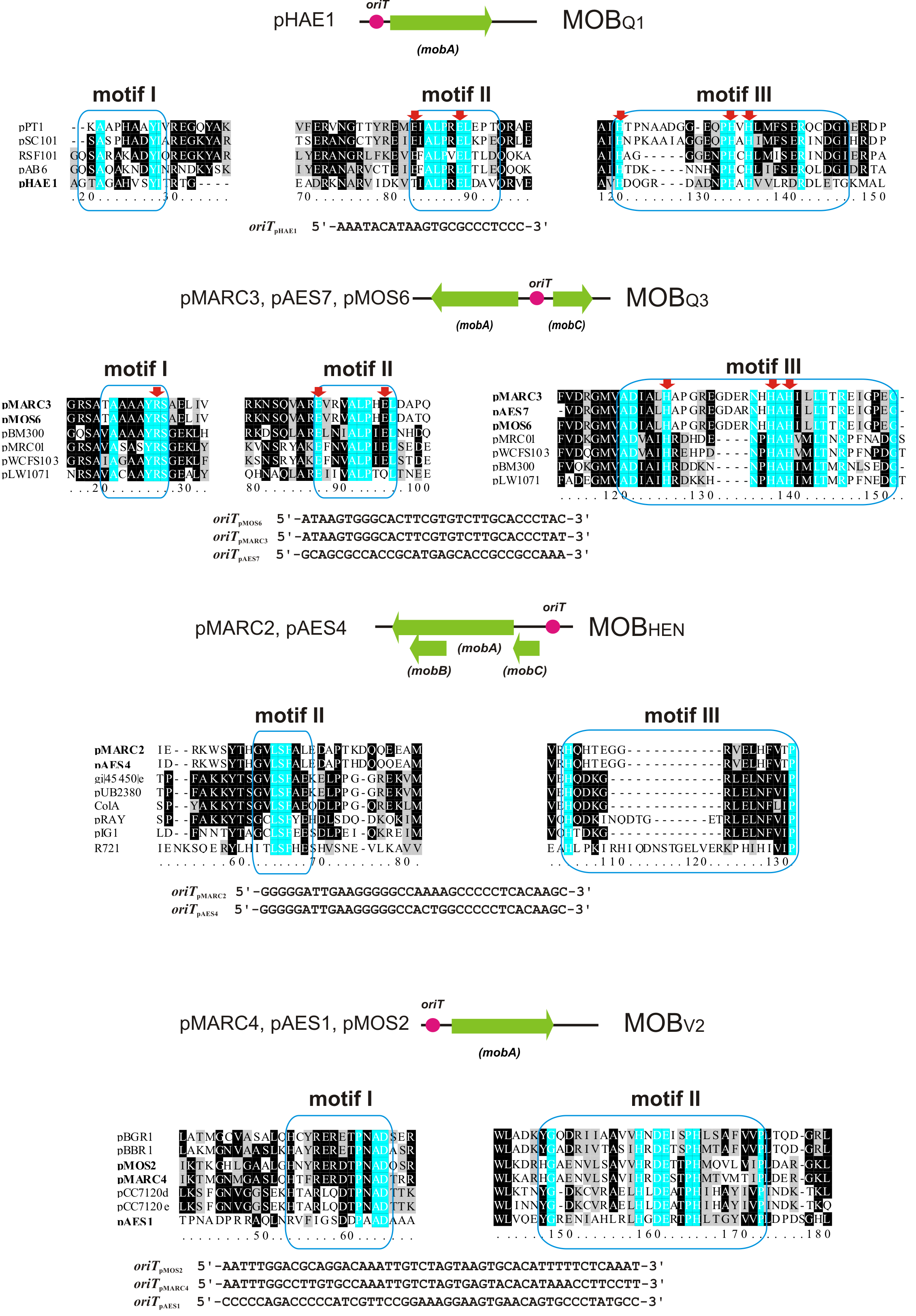

Supplement: Figure S2 — Comparison of sequence motifs identified in relaxases encoded within MOB modules of the Paracoccus spp. plasmids analyzed in this study. The conserved motifs identified within the relaxase (MobA) proteins of analyzed plasmids were present in a form of alignments. Conserved amino acids, characteristic for each motif (according to Francia et al. [31]; Garcillan-Barcia et al. [30]), were shown against the blue background. Other conserved amino acids common in more than 50% of analyzed sequences are shown against black background, and those common in less than 50% have gray background. For the alignments additional MobA sequences of various mobilization plasmids, classified into appropriate category (according Francia et al. [31]; Garcillan-Barcia et al. [30]) were used. (TIF) [file pone.0080258.s002.tif]

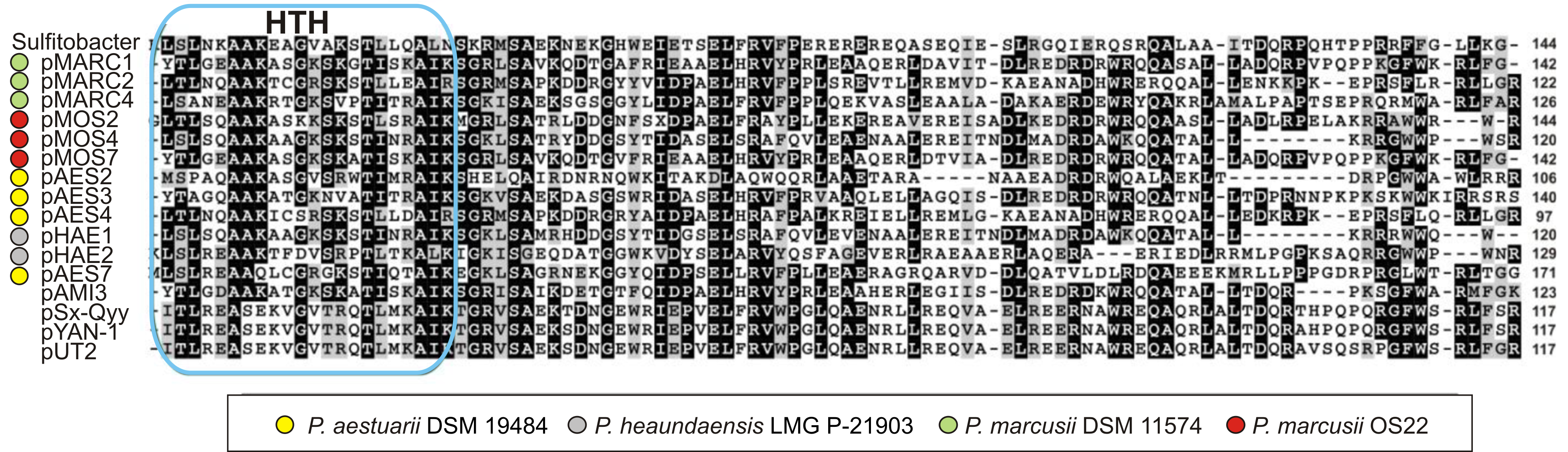

Supplement: Figure S3 — Multiple alignment of amino acid sequences of Exc1-like proteins encoded by Paracoccus spp. plasmids analyzed in this study. For the alignment the Exc1-like proteins of the following plasmids were used: pMARC1, pMARC2, pMARC4 of P. marcusii DSM 11574, pMOS2, pMOS4, pMOS7 of P. marcusii OS22, pAES2, pAES3, pAES4, pAES7 of P. aestuarii DSM 19484, pHAE1, pHAE2 of P. heaundaensis LMG P-21903, pAMI3 of P. aminophilus JCM 7686 (YP_003305342), pSX-Qyy of Sphingobium xenophagum QYY (sequence distinguished in this work), pYAN-1 of Sphingobium yanoikuyae JCM 7371 (sequence distinguished in this work), pUT2 of Sphingobium japonicum UT26S (YP_003550321), as well as protein sequence annotated within a contig of an unfinished genomic project of Sulfitobacter sp. NAS-14.1 (ZP_00964870). Amino acids identical in at least 50% of the analyzed sequences are shown against a black background, while those common to at least 15% of the analyzed sequences have a gray background. The HTH motifs were distinguished by blue frame. (TIF) [file pone.0080258.s003.tif]
