# Supplementary material for: Plasmids of Carotenoid-Producing Paracoccus spp. (Alphaproteobacteria) - Structure, Diversity and Evolution
Source: PLoS One. 2013 Nov 8;8(11):e80258. doi: 10.1371/journal.pone.0080258 (PMC3832669; doi:10.1371/journal.pone.0080258)
Supplement: Table S1 — ORFs located within the Paracoccus spp. plasmids analyzed in this study. (DOC) [file pone.0080258.s004.doc]

**Table S1. ORFs located within the *Paracoccus* spp. plasmids analyzed in this study.**

| **ORFs located within plasmid pAES1 of *P. aestuarii* DSM 19484** | | | | | | | |
| --- | --- | --- | --- | --- | --- | --- | --- |
| ORF no. | Coding region (bp) | Orientation | Protein size (aa) | Possible function | Best BLAST hits | | |
|  |  |  |  |  | Percentage identity (aa) | Organism | GenBank accession no. |
| 1 | 1-534 | → | 177 | replication protein (RepA) | 46 (70/153) | *Escherichia coli* | WP_000310024 |
| 2 | 709-2544 | → | 611 | mobilization protein A (MobA) | 38 (230/598) | *Zymomonas mobilis* subsp. mobilis ATCC 10988 | YP_001569033 |
| **ORFs located within plasmid pAES2 of *P. aestuarii* DSM 19484** | | | | | | | |
| ORF no. | Coding region (bp) | Orientation | Protein size (aa) | Possible function | Best BLAST hits | | |
|  |  |  |  |  | Percentage identity (aa) | Organism | GenBank accession no. |
| 1 | 1-1020 | → | 339 | replication protein (RepA) | 81 (276/339) | *Paracoccus haeundaensis* (plasmid pHAE1) | YP_006961987 |
| 2 | 1038-1376 | → | 112 | site-specific recombinase | 29 (33/112) | *Sphingomonas* sp. PAMC 26617 | YP_006961983 |
| 3 | 1438-2394 | → | 318 | DNA-cytosine methyltransferase | 89 (284/318) | *Acidovorax* sp. JS42 (plasmid pAOVO02) | YP_974087 |
| 4 | 2394-3254 | → | 286 | type II DNA restriction endonuclease | 94 (265/283) | *Stenotrophomonas maltophilia* Ab55555 | ZP_18104334 |
| 5 | 3307-3987 | ← | 226 | hypothetical protein | 31 (54/176) | *Maritimibacter alkaliphilus* HTCC2654 | ZP_01015242 |
| **ORFs located within plasmid pAES3 of *P. aestuarii* DSM 19484** | | | | | | | |
| ORF no. | Coding region (bp) | Orientation | Protein size (aa) | Possible function | Best BLAST hits | | |
|  |  |  |  |  | Percentage identity (aa) | Organism | GenBank accession no. |
| 1 | 1-1014 | → | 337 | replication protein (RepA) | 72 (246/341) | *Paracoccus pantotrophus* (plasmid pWKS1) | NP_690579 |
| 2 | 1113-1559 | → | 148 | site-specific recombinase | 48 (68/142) | *Paracoccus aminophilus* (plasmid pAMI3) | YP_003305342 |
| 3 | 1980-3104 | ← | 374 | hypothetical protein | 20 (59/294) | *Anaeromyxobacter dehalogenans* 2CP-C | YP_466742 |
| 4 | 3348-3629 | ← | 93 | hypothetical protein | 64 (49/77) | *Thiomicrospira crunogena* XCL-2 | YP_390638 |
| 5 | 3801-4088 | ← | 95 | ParE-like protein | 93 (77/83) | *Paracoccus aestuarii* (plasmid pAES7) | YP_006965315 |
| 6 | 4085-4333 | ← | 82 | CopG-like protein | 98 (80/82) | *Paracoccus aestuarii* (plasmid pAES7) | YP_006965314 |
| 7 | 4920-5234 | ← | 104 | hypothetical protein | 44 (21/48) | *Cystobacter fuscus* | WP_002630566 |
| **ORFs located within plasmid pAES4 of *P. aestuarii* DSM 19484** | | | | | | | |
| ORF no. | Coding region (bp) | Orientation | Protein size (aa) | Possible function | Best BLAST hits | | |
|  |  |  |  |  | Percentage identity (aa) | Organism | GenBank accession no. |
| 1 | 1-735 | → | 244 | replication protein (RepA) | 55 (127/233) | *Acidiphilium multivorum* AIU301 (plasmid pACMV6) | YP_004277313 |
| 2 | 806-1198 | → | 130 | site specific recombinase | 36 (50/137) | *Paracoccus aminophilus* (plasmid pAMI3) | YP_003305342 |
| 3 | 1216-2166 | ← | 316 | hypothetical protein | 51 (158/312) | *Methylobacterium extorquens* DM4 | YP_003068102 |
| 4 | 2978-3385 | ← | 135 | MobB | 25 (22/87) | *Bacteroides ovatus* | WP_004298967 |
| 5 | 2616-4115 | ← | 499 | mobilization protein A (MobA) | 46 (237/512) | *Oceaniovalibus guishaninsula* JLT2003 | ZP_11147119 |
| 6 | 4105-4425 | ← | 106 | mobilization protein C | 59 (61/104) | *Oceaniovalibus guishaninsula* JLT2003 | ZP_11147118 |
| 7 | 4574-4906 | → | 110 | hypothetical protein | 56 (46/82) | *Paracoccus* sp. TRP | ZP_08666545 |
| 8 | 4946-5221 | → | 91 | HigB-like protein (toxin) | 65 (59/91) | *Bartonella* sp. DB5-6 | ZP_10445876 |
| 9 | 5232-5531 | → | 99 | HigA-like protein (antidote) | 65 (63/97) | *Bradyrhizobium* sp. YR681 | ZP_10582436 |
| **ORFs located within plasmid pAES7 of *P. aestuarii* DSM 19484** | | | | | | | |
| ORF no. | Coding region (bp) | Orientation | Protein size (aa) | Possible function | Best BLAST hits | | |
|  |  |  |  |  | Percentage identity (aa) | Organism | GenBank accession no. |
| 1 | 1-576 | → | 191 | replication protein (RepA) | 62 (114/185) | *Sphingobium xenophagum* QYY (plasmid pSx-Qyy) | YP_195758 |
| 2 | 579-1112 | ← | 177 | site-specific recombinase | 51 (25/49) | *Paracoccus aestuarii* (plasmid pAES4) | YP_006963009 |
| 3 | 1390-1638 | → | 82 | CopG-like protein | 98 (80/82) | *Paracoccus aestuarii* (plasmid pAES3) | YP_006961715 |
| 4 | 1635-2105 | → | 156 | ParE-like protein | 93 (77/83) | *Paracoccus aestuarii* (plasmid pAES3) | YP_006961714 |
| 5 | 2492-5029 | → | 845 | hypothetical protein | 56 (293/520) | *Rhodobacter sphaeroides* ATCC 17025 (plasmid pRSPA02) | YP_001170161 |
| 6 | 5026-6327 | → | 433 | hypothetical protein | 68 (257/380) | *Rhodobacter sphaeroides ATCC 17025* (plasmid pRSPA02) | YP_001170162 |
| 7 | 6275-6895 | → | 206 | hypothetical protein | 63 (116/183) | Rhodobacter sphaeroides ATCC 17025 (plasmid pRSPA02) | YP_001170162 |
| 8 | 7173-8309 | → | 378 | UDP-galactose mutase | 82 (314/381) | *Paracoccus sp. TRP* | ZP_08663372 |
| 9 | 8494-9039 | → | 181 | nitroreductase | 49 (82/166) | *Rhodobacter sphaeroides ATCC 17025* (plasmid pRSPA02) | YP_001170158 |
| 10 | 9086-9457 | → | 123 | transposase | 94 (115/122) | *Ketogulonicigenium vulgare* Y25 (plasmid pYP1) | YP_003943981 |
| 11 | 9454-9843 | → | 129 | transposase | 96 (124/129) | *Rhodobacter* sp. AKP1 | ZP_19201186 |
| 12 | 10215-10967 | ← | 250 | mobilization protein A (MobA) | 91 (177/195) | *Paracoccus marcusii* (plasmid pMOS6) | YP_006964834 |
| 13 | 11097-11462 | → | 121 | hypothetical protein | 68 (30/44) | *Paracoccus marcusii* (plasmid pMOS6) | YP_006964836 |
| 14 | 11616-11912 | ← | 98 | toxin-antitoxin system protein | 70 (69/99) | *Xylella fastidiosa* 9a5c | NP_298991 |
| 15 | 11916-12212 | ← | 98 | toxin-antitoxin system protein | 69 (67/97) | *Xylella fastidiosa* Temecula1 | NP_779380 |
| **ORFs located within plasmid pHAE1 of *P. haeundaensis* LG P- 21903** | | | | | | | |
| ORF no. | Coding region (bp) | Orientation | Protein size (aa) | Possible function | Best BLAST hits | | |
|  |  |  |  |  | Percentage identity (aa) | Organism | GenBank accession no. |
| 1 | 1-1020 | → | 339 | replication protein (RepA) | 81 (276/339) | *Paracoccus aestuarii* (plasmid pAES2) | YP_006961982 |
| 2 | 1038-1406 | → | 122 | site-specific recombinase | 46 (54/117) | *Paracoccus marcusii* (plasmid pMOS7) | YP_006962997 |
| 3 | 1907-2170 | ← | 87 | hypothetical protein | 39 (26/66) | *Parvibaculum lavamentivorans* DS-1 | YP_001411399 |
| 4 | 3564-4994 | → | 476 | mobilization protein A (MobA) | 53 (102/194) | *Stappia aggregata* IAM 12614 | ZP_01551342 |
| **ORFs located within plasmid pHAE2 of *P. haeundaensis* LG P- 21903** | | | | | | | |
| ORF no. | Coding region (bp) | Orientation | Protein size (aa) | Possible function | Best BLAST hits | | |
|  |  |  |  |  | Percentage identity (aa) | Organism | GenBank accession no. |
| 1 | 1-984 | → | 327 | replication protein (RepA) | 60 (148/248) | *Sphingobium yanoikuyae* XLDN2-5 | ZP_09907537 |
| 2 | 981-1385 | → | 134 | site-specific recombinase | 41 (58/142) | *Sulfitobacter* sp. DFL14 (plasmid pDFL14-10) | YP_006963364 |
| 3 | 1638-2093 | → | 151 | hypothetical protein | 67 (92/138) | *Sphingomonas* sp. SKA58 | ZP_01304965 |
| 4 | 2422-3843 | ← | 473 | hypothetical protein | 46 (223/483) | *Wohlfahrtiimonas chitiniclastica* SH04 | ZP_21241726 |
| 5 | 4238-5536 | → | 432 | hypothetical protein | 53 (114/216) | *Paracoccus methylutens* (plasmid pMTH4) | AAQ19960 |
| **ORFs located within plasmid pMARC1 of *P. marcusii* DSM 11574** | | | | | | | |
| ORF no. | Coding region (bp) | Orientation | Protein size (aa) | Possible function | Best BLAST hits | | |
|  |  |  |  |  | Percentage identity (aa) | Organism | GenBank accession no. |
| 1 | 369-1118 | → | 249 | replication protein (RepA) | 97  (242/249) | *Paracoccus marcusii* OS22 (plasmid pMOS7) | YP_006962996 |
| 2 | 1220-1663 | → | 147 | entry exclusion protein 1 (Exc1) | 89  (131/147) | *P. marcusii* OS22 (plasmid pMOS7) | YP_006962997 |
| 3 | 1782-2177 | → | 131 | hypothetical protein | - | no similarity found | - |
| 4 | 2242-3411 | ← | 389 | GTP pyrophosphokinase; RelA/SpoT domain-containing protein | 47  (125/264) | *Idiomarina loihiensis* L2TR | YP_155038 |
| 5 | 3779-4042 | ← | 87 | antitoxin of toxin-antitoxin system | 99  (86/87) | *P. marcusii* OS22 (plasmid pMOS7) | YP_006963001 |
| 6 | 4026-4310 | ← | 94 | toxin of toxin-antitoxin system | 98  (91/93) | *P. marcusii* OS22 (plasmid pMOS7) | YP_006963002 |
| **ORFs located within plasmid pMARC2 of *P. marcusii* DSM 11574** | | | | | | | |
| ORF no. | Coding region (bp) | Orientation | Protein size (aa) | Possible function | Best BLAST hits | | |
|  |  |  |  |  | Percentage identity (aa) | Organism | GenBank accession no. |
| 1 | 240-977 | → | 245 | replication protein (RepA) | 92  (224/244) | *Paracoccus aestuarii* DSM 19484  (plasmid pAES4) | YP_006963008 |
| 2 | 1126-1437 | → | 103 | entry exclusion protein 1 (Exc1) | 73  (75/103) | *P. aestuarii* DSM 19484  (plasmid pAES4) | YP_006963009 |
| 3 | 1786-3033 | → | 415 | ATPase, SMC domain-containing protein | 42  (175/416) | *Microcoleus* sp. PCC 7113 | YP_007120442 |
| 4 | 3033-3668 | → | 211 | hypothetical protein | 33  (49/147) | *Microcoleus* sp. PCC 7113 | YP_007120441 |
| 5 | 3782-5281 | ← | 499 | mobilization protein A (MobA) | 83  (416/499) | *P. aestuarii* DSM 19484  (plasmid pAES4) | YP_006963011 |
| 6 | 5271-5591 | ← | 106 | mobilization protein C (MobC) | 90  (95/106) | *P. aestuarii* DSM 19484  (plasmid pAES4) | YP_006963013 |
| **ORFs located within plasmid pMARC3 of *P. marcusii* DSM 11574** | | | | | | | |
| ORF no. | Coding region (bp) | Orientation | Protein size (aa) | Possible function | Best BLAST hits | | |
|  |  |  |  |  | Percentage identity (aa) | Organism | GenBank accession no. |
| 1 | 1702-2268 | → | 188 | replication protein (RepA) | 62  (112/181) | *P. aestuarii* DSM 19484  (plasmid pAES7) | YP_006965312 |
| 2 | 2370-2981 | → | 203 | partitioning protein A (ParA) | 44  (88/202) | *Methylobacterium radiotolerans* JCM 2831 (plasmid pMRAD03) | YP_001776801 |
| 3 | 2978-3244 | → | 88 | hypothetical protein | - | no similarity found | - |
| 4 | 3454-5169 | ← | 569 | hypothetical protein | 45  (152/340) | *Microcoleus sp.* PCC 7113 | YP_007121045 |
| 5 | 6258-7286 | → | 342 | transposase | 85  (290/341) | *Brucella inopinata* BO1 | ZP_07478924 |
| 6 | 7885-8982 | ← | 365 | mobilization protein A (MobA) | 94  (289/308) | *P. marcusii* OS22 (plasmid pMOS6) | YP_006964834 |
| 7 | 9140-9391 | → | 83 | mobilization protein C (MobC) | 98  (81/83) | *P. marcusii* OS22 (plasmid pMOS6) | YP_006964835 |
| 8 | 9388-9948 | → | 186 | hypothetical protein | 73  (68/93) | *P. marcusii* OS22 (plasmid pMOS6) | YP_006964836 |
| 9 | 10137-10385 | → | 82 | toxin of toxin-antitoxin system | 80  (66/82) | *Rhodobacter sphaeroides* 2.4.1 | YP_352820 |
| 10 | 10382-10672 | → | 96 | antitoxin of toxin-antitoxin system | 81  (72/89) | *Rhodobacter sphaeroides* WS8N | ZP_08412517 |
| **ORFs located within plasmid pMARC4 of *P. marcusii* DSM 11574** | | | | | | | |
| ORF no. | Coding region (bp) | Orientation | Protein size (aa) | Possible function | Best BLAST hits | | |
|  |  |  |  |  | Percentage identity (aa) | Organism | GenBank accession no. |
| 1 | 277-1299 | → | 340 | replication protein (RepA) | 63  (213/339) | *P. aestuarii* DSM 19484  (plasmid pAES3) | YP_006961710 |
| 2 | 1334-1744 | → | 136 | entry exclusion protein 1 (Exc1) | 62  (84/135) | *Citreicella sp.* 357 | ZP_10022074 |
| 3 | 1790-2008 | → | 72 | antitoxin of toxin-antitoxin system | 65  (47/72) | *Dinoroseobacter shibae* DFL 12 (plasmid pDSHI01) | YP_001541878 |
| 4 | 2005-2331 | → | 108 | toxin of toxin-antitoxin system | 64  (68/107) | *Rhodospirillum photometricum* DSM 122 | YP_005417350 |
| 5 | 2716-3897 | ← | 393 | acyltransferase | 65  (237/363) | *Rhodobacter sphaeroides* 2.4.1 (plasmid D) | YP_345398 |
| 6 | 4564-5247 | → | 227 | PA-phosphatase-like phosphoesterase | 34  (74/216) | *Methylobacterium sp.* 4-46 | YP_001767744 |
| 7 | 5750-6769 | ← | 339 | acyltransferase | 30  (103/346) | *Sphingobium sp.* SYK-6 | YP_004836036 |
| 8 | 6808-7131 | → | 107 | putatitve replication protein | 60  (33/55) | *Pseudomonas stutzeri* 2A54 (plasmid p2A54) | AEP40493 |
| 9 | 7160-7636 | → | 158 | hypothetical protein | 31  (34/110) | *Brachybacterium paraconglomeratum* LC44 | ZP_10096259 |
| 10 | 8053-9384 | → | 443 | polysaccharide biosynthesis protein | 60  (262/434) | *Mesorhizobium ciceri* biovar biserrulae WSM1271 | YP_004139455 |
| 11 | 9420-10445 | → | 341 | phosphoribosyltransferase | 63  (212/338) | *Mesorhizobium australicum* WSM2073 | YP_007302113 |
| 12 | 10471-11352 | → | 293 | dolichyl-phosphate mannose synthase related protein | 45  (133/293) | *Novosphingobium pentaromativorans* US6-1 | ZP_09191429 |
| 13 | 11450-12472 | ← | 340 | glycosyltransferase | 36  (110/306) | *Hirschia baltica* ATCC 49814 | YP_003060525 |
| 14 | 12643-13818 | ← | 391 | UDP-glucose 6-dehydrogenase | 72  (281/388) | *Roseobacter sp.* SK209-2-6 | ZP_01756788 |
| 15 | 14000-15289 | → | 429 | mobilization protein A (MobA) | 60  (228/382) | *Paracoccus pantotrophus* DSM 11072 (plasmid pWKS1) | NP_690578 |
| **ORFs located within plasmid pMOS2 of *P. marcusii* OS22** | | | | | | | |
| ORF no. | Coding region (bp) | Orientation | Protein size (aa) | Possible function | Best BLAST hits | | |
|  |  |  |  |  | Percentage identity (aa) | Organism | GenBank accession no. |
| 1 | 1-558 | → | 185 | replication protein (RepA) | 67 (112/166) | *Sphingobium xenophagum* QYY (plasmid pSx-Qyy) | YP_195758 |
| 2 | 620-1066 | → | 148 | site-specific recombinase | 45 (65/144) | *Paracoccus haeundaensis* (plasmid pHAE1) | YP_006961988 |
| 3 | 2190-2936 | → | 248 | hypothetical protein | 38 (32/85) | *Thioalkalivibrio sp.* K90mix | YP_003460377 |
| 4 | 3706-4347 | ← | 213 | hypothetical protein | 67 (142/213) | uncultured bacterium | EKD60510 |
| 5 | 4652-5929 | → | 425 | relaxase | 61 (150/245) | *Paracoccus pantotrophus* (plasmid pWKS1) | NP_690578 |
| **ORFs located within plasmid pMOS6 of *P. marcusii* OS22** | | | | | | | |
| ORF no. | Coding region (bp) | Orientation | Protein size (aa) | Possible function | Best BLAST hits | | |
|  |  |  |  |  | Percentage identity (aa) | Organism | GenBank accession no. |
| 1 | 1-552 | → | 183 | replication protein (RepA) | 54 (94/174) | *Bartonella taylorii* | WP_004861374 |
| 2 | 723-1181 | ← | 152 | hypothetical protein | 33 (27/81) | *Nitratiruptor* sp. SB155-2 | YP_001356124 |
| 3 | 1381-1668 | ← | 95 | hypothetical protein | 38 (15/39) | *Bacteroides coprosuis* | WP_006744552 |
| 4 | 1822-2919 | ← | 365 | alcohol dehydrogenase | 99 (360/362) | *Sphingomonas echinoides* ATCC 14820 | ZP_10341701 |
| 5 | 3291-3446 | → | 51 | hypothetical protein | 72 (33/46) | *Sphingomonas* sp. LH128 | ZP_10870845 |
| 6 | 3452-3931 | → | 159 | transposase | 74 (107/144) | *Acetobacter pasteurianus* | WP_003626917 |
| 7 | 3987-4340 | → | 117 | hypothetical protein | 30 (23/77) | *Pseudomonas stutzeri* DSM 10701 | YP_006522958 |
| 8 | 4511-5599 | ← | 362 | mobilization protein A (MobA) | 79 (232/294) | *Paracoccus aminophilus* (plasmid pAMI3) | YP_003305343 |
| 9 | 5756-6007 | → | 83 | mobilization protein C (MobC) | 86 (71/83) | *Paracoccus aminophilus* (plasmid pAMI3) | YP_003305344 |
| 10 | 6261-6566 | → | 101 | hypothetical protein | 49 (38/78) | *Sulfitobacter* sp. NAS-14.1 | ZP_00964866 |
| 11 | 6573-6908 | ← | 111 | Ata-like protein | 64 (68/107) | *Agrobacterium* sp. ATCC 31749 | WP_006312741 |
| 12 | 6905-7270 | ← | 121 | Tad-like protein | 68 (81/119) | delta proteobacterium MLMS-1 | ZP_01287697 |
| **ORFs located within plasmid pMOS7 of *P. marcusii* OS22** | | | | | | | |
| ORF no. | Coding region (bp) | Orientation | Protein size (aa) | Possible function | Best BLAST hits | | |
|  |  |  |  |  | Percentage identity (aa) | Organism | GenBank accession no. |
| 1 | 1-750 | → | 249 | replication protein (RepA) | 47 (113/241) | *Laribacter hongkongensis* (plasmid pHLHK19) | ABC70160 |
| 2 | 870-1313 | → | 147 | site-specific recombinase | 59 (86/147) | *Paracoccus aminophilus* (plasmid pAMI3) | YP_003305342 |
| 3 | 1650-1997 | → | 115 | hypothetical protein | - | - | - |
| 4 | 2231-3679 | ← | 482 | retron type reverse transciptase | 27 (93/344) | *Geitlerinema* sp. PCC 7407 | YP_007109636 |
| 5 | 3868-4137 | ← | 89 | hypothetical protein | 34 (28/82) | *Rhizobium etli* CIAT 652 | YP_001979209 |
| 6 | 4265-4528 | ← | 87 | antitoxin of toxin-antitoxin system | 76 (66/87) | *Gluconacetobacter* sp. SXCC-1 | ZP_08314291 |
| 7 | 4512-4796 | ← | 94 | toxin of toxin-antitoxin system | 75 (68/91) | *Desulfomicrobium baculatum* DSM 4028 | YP_003157267 |
