# Supplementary material for: Plasmids of Carotenoid-Producing Paracoccus spp. (Alphaproteobacteria) - Structure, Diversity and Evolution
Source: PLoS One. 2013 Nov 8;8(11):e80258. doi: 10.1371/journal.pone.0080258 (PMC3832669; doi:10.1371/journal.pone.0080258)
Supplement: Table S2 — Oligonucleotide primers used in this study. (DOCX) [file pone.0080258.s005.docx]

| **Primer** | **Sequence (5’→3’)** | **Target DNA** |
| --- | --- | --- |
| **Primer pairs used for the PCR amplification of hybridization probes** | | |
| repMOS7L | GCTTCATCTGGCTGGCACTT | REP pMOS7 |
| repMOS7R | ACGTCACGATGCACAACACC |  |
| repMOS2L | AATCCATCTGCGGCGAGACT | REP pMOS2 |
| repMOS2R | TGCCAGTGTGGAAGGAAGGT |  |
| repMOS4L | CGCAATGCGCCTAGTCTTCA | REP pMOS4 |
| repMOS4R | GCAACTGCCACTGTCTCTGT |  |
| P2REPL | CCTCAAGCTGATGCACCTGC | REP pAES2 |
| P2REPR | AAGCCTGCTCGATACCACCA |  |
| P3REPL | GGAGATTGCAGCGGCTCTTG | REP pHAE2 |
| P3REPR | CGATCATCTTCGGACAGAGC |  |
| P4REPL | CCTGCGCAAGCTGTTCATCG | REP pHAE1 |
| P4REPR | CTGCTGAACAGCTCCTCGAT |  |
| P6REPL | GAAGCGATCTCGGATGAAGA | REP pAES1 |
| P6REPR | TTCCGAGTCCACAACGTCGT |  |
| P1REPL | CACCTACATGCAGAATGCTC | REP pAES3 |
| P1REPR | TCGGCAGAAGTCCTCGAACA |  |
| REPAES7L | AATCTCCGGTTGGCGCTCAG | REP pAES7 |
| REPAES7R | ATCCAGTCGCTTGCCGGTCT |  |
| REPAES4L | GACTTCTTCGGAGGGGGCAA | REP pAES4 |
| REPAES4R | TGCGCATGGTTATGCACGTT |  |
| REPMAR5L | CCTCAGATGACGGCATAGCA | REP pMAR5 |
| REPMAR5R | GCGATCAATACCGGAGGTCA |  |
| **Primer pairs used for the PCR amplification of REP modules** | | |
| KMOS6L | ATAATCTACAGCAGCCGGCA | REP pMOS6 (used for construction of pKRP-DIY_pAES7_) |
| KMOS6SPE | agaactagtCAGTACGTTCGATGCGCCAC |  |
| LpAES1Kp | taggtaccTTTCGACAACGACCCCGTAG | REP pAES7 (construction of shuttle vector pVIV7) |
| RpAES1Xb | gctctagaAATGGCCGATTCCACAAGGT |  |

**Table S2. Oligonucleotide primers used in this study.**
